# Simultaneous inhibition of TRIM24 and TRIM28 sensitises prostate cancer cells to antiandrogen therapy, decreasing VEGF signalling and angiogenesis

**DOI:** 10.1002/1878-0261.70065

**Published:** 2025-05-24

**Authors:** Damien A. Leach, Nilesh Chatterjee, Kellie Spahr, Gilberto Serrano de Almeida, Anabel Varela‐Carver, Taimur T. Shah, Mathias Winkler, Hashim U. Ahmed, Charlotte L. Bevan

**Affiliations:** ^1^ Division of Cancer, Imperial Centre for Translational & Experimental Medicine Imperial College London UK; ^2^ St George's University of London UK; ^3^ University of Michigan Ann Arbor MI USA; ^4^ University of Pittsburgh PA USA; ^5^ Imperial Urology, Division of Surgery Imperial College Healthcare NHS Trust London UK; ^6^ Imperial Prostate, Department of Surgery and Cancer, Faculty of Medicine Imperial College London UK

**Keywords:** androgen receptor, angiogenesis, coregulator, therapy, TRIM proteins, VEGF

## Abstract

Castrate‐resistant prostate cancer (CRPC) is a likely outcome of hormone treatment for advanced prostate cancer. Although no longer dependent on androgen levels, CRPC remains driven by the androgen receptor (AR). One proposed progression mechanism is altered repertoires of coregulator proteins possessing the ability to alter AR activity. Increased expression of tripartite motif‐containing 24 (*TRIM24*) and *TRIM28*—two members of a distinct bromodomain‐containing subfamily of Tripartite motif (TRIM) coregulators—occurs in CRPC. Endogenous TRIM24 and TRIM28 interact with each other and AR, bind to chromatin and regulate genes such as the angiogenic factor vascular endothelial growth factor A (*VEGFA*) and oncogene *MYC*. Silencing of *TRIM24* and *TRIM28* simultaneously, but not either alone, sensitised CRPC model cell lines to the antiandrogen enzalutamide and bicalutamide. This re‐sensitisation to antiandrogen therapeutics could then be reversed by addition of VEGF. Furthermore, both TRIM24 and TRIM28 expression associated with angiogenesis signatures in tumour samples, and conditioned media from TRIM24 and TRIM28‐silenced cancer cells inhibited endothelial cell proliferation and formation of vascular tube structures. Our data suggest that TRIM24 and TRIM28 proteins interact, in gene‐specific manners, to regulate AR activity, increase VEGF signalling and angiogenesis, and that targeting these coregulators may increase the effectiveness of antiandrogen therapy.

## Graphical abstract

TRIM24 and TRIM28 are androgen receptor (AR) coregulators which exhibit increased expression with cancer progression. Both TRIM24 and TRIM28 combine to influence the response of castrate‐resistant prostate cancer (CRPC) cells to AR inhibitors by mediating AR signalling, regulation of MYC and upregulating VEGF to promote angiogenesis.

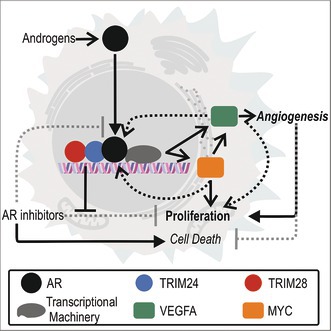

**Abbreviations**
ADT: androgen deprivation therapy
AR: androgen receptor
ARGs: androgen‐regulated genes
BIC: bicalutamide
ChIP: chromatin Immunoprecipitation
CM: conditioned media
CoIP: co‐immunoprecipitation
CRPC: castrate‐resistant prostate cancer
DHT: dihydrotestosterone
ENZ: enzalutamide
FFPE: formalin‐fixed paraffin embedded
IF: immunofluorescence
IHC: immunohistochemistry
NR: nuclear receptor
PCa: prostate cancer
TMA: tissue microarray
TRIM: tripartite motif containing
VC: vehicle control
VI: vascularisation index signature

## 1 Introduction

Prostate cancer (PCa) patients with advanced or metastatic disease are commonly treated with hormonal therapies aimed at blocking the signalling of the androgen receptor (AR). AR signalling is pivotal to PCa development and progression. Translocating from the cytoplasm to the nucleus upon ligand activation (androgen binding), the AR drives transcriptional processes involved in prostate differentiation but, in cancer, also drives proliferative pathways that mediate cancer progression. Given this critical role of AR in PCa biology, hormonal therapies include androgen deprivation therapy (ADT) to block androgen production and antiandrogens such as bicalutamide, enzalutamide and darolutamide to directly inhibit AR activity. Blocking AR signalling is initially effective in reducing tumour burden, however the disease will ultimately re‐emerge as lethal castrate‐resistant PCa (CRPC) [1]. Importantly, in CRPC, AR signalling remains active, this can also occur through intra‐tumoral androgen production, AR amplification and AR splice variants [2], but here we will focus on the influence of coregulators in CRPC progression.

As well as the AR itself, another key component of AR signalling is the group of proteins termed coregulators, which comprises currently over 300 proteins which either amplify (co‐activator) or reduce (corepressor) AR activity [3]. Coregulators exert their effects largely in two ways. Firstly, they can interact with and modify histones to affect interactions with DNA, regulating chromatin remodelling/accessibility and recruitment of the transcriptional machinery. Secondly, coregulators can affect the dynamic activity of the AR protein, influencing stability, ligand‐binding, intracellular movement and interaction with other proteins [4, 5, 6]. Changes in coregulator expression are believed to be one of the mechanisms by which PCa can survive antiandrogen therapy and progress to CRPC [3, 7, 8]. Specifically, the alteration of coregulators in CRPC is suggested to allow AR to be recruited to or remain active on DNA despite lack of circulating androgens and/or presence of antiandrogens and to regulate key genes in the absence of ligand [3, 9].

Tripartite motif‐containing (TRIM) proteins are one family of coregulators [10]. Within this, a small subgroup known as Class C‐VI family consisting of TRIM24, TRIM28 and TRIM33 [10, 11, 12] are unique among TRIM proteins in that while they contain RING‐finger, B‐box and coiled‐coil domains, they also contain a PHD‐finger domain and bromodomain, which are able to recognise and bind to acetylated and methylated histones [10, 13, 14]. They are also frequently found to be dysregulated in CRPC; while TRIM33 has been reported as downregulated, TRIM24 and TRIM28 emerged from systematic analysis of the literature as coregulators frequently upregulated in CRPC [15]. Both TRIM24 (TIF1α) and TRIM28 (TIF1β/KAP1) interact directly with AR: TRIM24 interacts with the C‐terminal transcription activation function (AF‐2) domain [16, 17], and TRIM28 interacts with another region important for transcriptional activation, the Tau1 domain in the N terminus [18]. Both TRIM24 and TRIM28 have been reported to be able to read histone marks/status via the PHD and bromodomain domains, as well as being able to modify acetylation and methylation status of histones [13, 19, 20, 21]. TRIM24 is a known co‐activator that can interact with histones (H3K4/H3K23) [16, 21, 22], while TRIM28 has been described as having repressor function and interacting with a number of ligand‐dependent corepressor proteins [23, 24, 25], but its expression is also associated with increased AR activity [18, 25]. Furthermore, the expression of both is regulated by AR in a potential feedback loop [22, 25]. Here, we explore the interactions between TRIM24, TRIM28 and AR in androgen signalling and CRPC. We find a role for TRIM24 and TRIM28 acting together to mediate AR‐driven PCa cell survival, by upregulating proliferation, downregulating caspase activity and potentially inducing angiogenesis through VEGFA control.

## 2 Materials and methods

### 2.1 Cell lines, culture and proliferation assays

LNCaP (RRID:CVCL_0395), C4‐2B (RRID:CVCL_4784), DU145 (RRID:CVCL_0105), PC3 (RRID:CVCL_0035), 22Rv1 (RRID:CVCL_1045) cell lines were previously purchased from ATCC, Manassas, VA, USA; these and 22Rv1 cells with no full‐length AR (22Rv1‐ARflKO (RRID:CVCL_WK44), kind gift from Luke Gaughan, Newcastle University, UK, [26]), R1‐AD1 (RRID:CVCL_ZC60) and R1‐D567 (RRID:CVCL_ZC61) CWR‐R1 cell lines (kind gifts from Scott Dehm, University of Minnesota, USA, [27]) were routinely grown in RPMI media (Gibco, Carlsbad, CA, USA) supplemented with 10% FBS (F9665, Sigma‐Aldrich, St Louis, MA, USA). In assays where DHT activity is measured, cells were plated and grown in Phenol‐Red‐Free (PRF) RPMI media supplemented with 5% Dextran Coated Charcoal stripped FBS (02‐48‐850, First Link, Wolverhampton, UK). In experiments where enzalutamide was solely investigated, RPMI with full FBS was used. Cell lines were authenticated with short tandem repeat analysis (Eurofins, Luxembourg city, Luxembourg) and routinely checked for mycoplasma contamination. HUVEC cells (Lonza C2519, Basel, Switzerland) were maintained in EGM Endothelial Medium BlueKit with supplements (Lonza, CC‐3121 CC‐4133).

Cell numbers were measured using the Crystal Violet assay (C0775, Sigma‐Aldrich) or MTT reagent (M6494, Sigma‐Aldrich) as per previous studies [28]. Briefly, in 96‐well plates, PC3 cells were plated at 5 × 10^5^ cells per well, while all other cell lines were plated at 3 × 10^4^. In experiments testing the effects of DHT, cells were plated into 96‐well plated at 1 × 10^3^ cells per well in 5%DCC‐FBS PRF‐RPMI. Treatment consisted of fresh media supplemented with DHT (A8380, Sigma‐Aldrich), enzalutamide (Enz, S1250‐SEL, Stratech Scientific, Cambridge, UK), bicalutamide (BIC, B9061, Sigma‐Aldrich) or vehicle control (VC, ethanol, DMSO, Sigma‐Aldrich). For crystal violet assays, cells were fixed with 1% formalin for 1 h, before washing with PBS, drying, staining with crystal violet (0.4%) for 1 h, washes with water, drying elution with acid and reading at 570 nm OD. For MTT, cells were incubated for 4 h with MTT media (5 mg per 10 mL media), then the dye was eluted with isopropanol solution and read at 570 nm OD (Tecan SUNRISE, LifeSciences, Männedorf, Switzerland).

For transfection studies, cells were transfected with 5 μm of pooled siRNA against TRIM24 or TRIM28 or negative control (Thermo Fisher Scientific, Waltham, MA, USA). In proliferation assays, transfection occur in 96‐well plates the day after seeded, with treatments added the day after, assay carried out as above. For assays evaluating the effect of enzalutamide or bicalutamide alone, cells were treated in full RPMI media with 10% FCS. For studies including VEGF, cells were treated with 10 ng·mL^−1^ of recombinant VEGF protein (PHC9394, Gibco Thermo Fisher). For RNA and protein collection, transfection occurred the day after plating, and treatment day the following day, then assay followed as below.

### 2.2 Expression analysis of genes in prostate disease

The expression of over 300 known nuclear receptor (NR) interacting proteins was assessed in three data sets GSE35988 (*N* = 226 patients) [29], GSE70770 (only patients with samples containing > 70% tumour, *N* = 68 patients) [30], GSE32269 (*N* = 51 PCa patient samples) [31]. Raw array data (GSE35988, GSE32269) or normalised/transformed data (GSE70770) were processed using AltAnalyzer [32], incorporating probeset/feature extraction, alignment, RMA array normalisation and batch effect removal with combat. Differential gene expression was determined as above a 0.5 log2 fold change between disease states with an adjusted *P*‐value (Benjamini–Hochberg) of < 0.05. LOG2 expression data were also transformed to *Z*‐Score for each gene (for each gene = (sample − mean)/stdev), individually within each cohort relative to all samples.

Analysis of genes potentially associated with *AR*, *TRIM24* and *TRIM28* was assessed by the following means. To assess AR in CRPC, AR‐ChIP data from models of CRPC (22Rv1 [33], C4‐2B [34], LNCaP‐abl [22] and LNCaP‐Bic‐resistant [35]) cell lines were compared to AR‐ChIP data from androgen‐sensitive LNCaP cells. Similar TRIM24 was assessed between LNCaP‐abl and LNCaP cells, and TRIM28 was assessed in LNCaP cells treated plus minus androgen. Binding was assessed with Cistrome, using the Snakemake analysis pipeline, read mapping/filtering/sorting using bwa then samtools, peaks called with MACS2 (*q* < 0.01) [36, 37]. Peaks with less than fivefold signal‐background ratios were filtered out. BETA analysis and creation of regulatory potential scores for each gene were used to identify genes related to the binding peaks [38]. Pathway analysis was conducted using shinygo [39].

Two vascularisation gene signatures—a 14 gene positive vascularisation signature (VI^+^) and a 22 gene negative vascularisation signature (VI^−^) [40] were assessed in GSE35988 (*N* = 226 patients), GSE70770 (*N* = 68 patients), GSE32269 (*N* = 51 PCa patient samples) and TCGA (*N* = 499 patients). An AR activity gene signature (27 genes [41]) and a CRPC‐AR activity gene signature (16 genes [42]) were also examined in the TCGA and MSKCC PCa cohorts. Associations between TRIM24, TRIM28, vascularisation signatures, AR signatures and TRIM‐targeted genes were assessed using IBM spss Statistics 24, NY, USA.

### 2.3 Patient‐derived explant (PDE) tissue culture

Collagen dental sponges (Surgispon, Aegis Lifesciences, Gujarat, India) were soaked in RPMI media supplemented with 5% FBS and 1 mm enzalutamide or equivalent vehicle control (VC) [43]. Tissue was collected from consenting patients and immediately transferred in ice‐cold RPMI. Tissue was carefully dissected into 1 mm^3^ cubes and placed onto presoaked sponges in 24‐well tissue culture plates. A further 500 μL of treatment‐containing media was added to each well. After 72‐h culture, tissue was collected in formalin for histological studies or snapped frozen for RNA analysis. Histological examination and gene expression confirmed the presence of cancer.

The study methodologies conformed to the standards set by the Declaration of Helsinki. Patient material was collected from Charing Cross Hospital (London, UK) between 2018 and 2023 under the ethical approval of Imperial College Healthcare Tissue Bank (REC 22/WA/0214) under the subcollection reference number Uro_MW_13_010. The use and methodology of human tissue experiments were approved under project id R18041. Samples were taken from patients undergoing diagnosis of high‐volume primary disease, with an age range between 55 and 88 years old. All sampling was undertaken after each subject confirmed understanding and was given written consent.

### 2.4 *In vivo* experiments

Work was conducted under the provisions of the Animals (Scientific Procedures) Act 1986 of the United Kingdom (HMSO, London, UK, 1990) and was approved by Imperial College Animal Welfare and Ethical Review Body. Studies were carried out under the project licence number 70/8705. In house transgenic mouse strain was used [44, 45], ARE‐Luc expressing *Pten* ^loxp/loxp^; *Pb*‐*Cre4* mice (PTEN deletion in prostate epithelial cells) with a C57BL/6J background (Jackson Laboratory, Bar Harbor, ME, USA). Mice were housed in a centralised animal facility at Imperial College London, in isolated ventilated cages under pathogen‐free conditions with humidity and temperature controlled, and exposed to 12‐h day/night cycling. 3–4 mice were housed per cage. All mice had access to food and water *ad libitum*, as well as bedding and nesting material. Male mice between 26 and 32 weeks old were treated with 6 mg·kg^−1^ IBET and/or 50 mg·kg^−1^ Enzalutamide (in 5%DMSO + 1%CMC + 0.1% P80 oral gavage) every day for 3 days, with weights and general health measured/observed over the course of experiments before being sacrificed. Tissue was harvested for RNA analysis with RT‐qPCR, and other samples were processed with FFPE for IHC analysis.

### 2.5 RT‐qPCR and immunoblot

*Into 6‐well plates*, cells were seeded at 5 × 10^5^. In experiments where DHT alone or in combination with enzalutamide was being tested, cells were then treated with combinations of DHT, enzalutamide and vehicle (ethanol and/or DMSO as appropriate) in PRF‐PRMI with DCC‐FCS. In experiments where only the effect of enzalutamide compared to vehicle control (VC) was being tested, the cells were treated with enzalutamide in RPMI with 10% FCS, with DMSO as used at the vehicle control. For RNA, cell treated for 24 h, and total RNA was prepared using the Monarch® kit according to the manufacturer's instructions (New England Biolabs, Ipswich, MA, USA), cDNA was created (RevertAid First Strand cDNA synthesis Kit, Thermo Fisher Scientific), and expression was measured with QuantStudio™ 7 (Applied Biosystems, Waltham, MA, USA), using SYBR green (Thermo Fisher Scientific) with primers detailed in Table S1. Primer efficiencies were calculated for all primers, and melt curves were run for all reactions. For protein, lysates were collected after 48 h treatments in RIPA buffer supplemented with protease inhibitors (1/200 μL, P8340, Sigma) and phosphatase inhibitors (1/200 μL, P5726, Sigma). Protein was mixed with SDS‐loading buffer and run on 10% gels. Protein was transferred to Immobilon‐P membranes (IPVH85R Sigma), that were blocked with skim milk for 2 h before immunostaining with antibodies against TRIM24 (1/1000 Santa Cruz, sc‐271266, Paso Robles, CA, USA), TRIM28 (1/1000 Invitrogen, 730029, Waltham, MA, USA), AR (1/2000 SantaCruz, sc‐7305x), MYC (1/1000 Abcam, ab32072, Cambridge, UK), FKBP5 (1/2000 SantaCruz, sc‐13983), VEGFA (1/500 Santa Cruz, sc‐7269), total caspase 3 (1/1000 Cell Signalling, 9662s, Danvers, MA, USA), cleaved caspase‐3 (1/1000 Cell Signalling, 9661S) and B‐Actin (1/3000 Abcam, ab6276). Primary antibodies were probed with peroxidase‐labelled secondary antibodies (Sigma A0545/A5278), detected with SuperSignal™ West Pico PLUS Chemiluminescent Substrate (Thermo Fisher) and imaged with iBright CL1500 (Thermo Fisher Scientific).

### 2.6 Co‐immunoprecipitation (CoIP)

Method adapted from published technique [46]. Briefly, LNCaP and 22RV1 cells were seeded in 10 cm plates at 1 × 10^6^ cells per plate and grown in PRF‐RPMI with DCC‐FCS until 80% confluence, 1 plate per IP and treatment. Cells were treated with 10 nm DHT for 4 h, then washed with PBS and lysed with lysis buffer (50 mm Tris/HCl pH 7.4, 150 mm NaCl, 1 mm EDTA, 2 mm EGTA, 0.25% CHAPS, plus protease inhibitors). Lysates were collected and centrifuged, with the supernatant incubated overnight at 4 °C with Protein G Dynabeads (10004D, Life Technologies) that had been precoated with 5 ng of antibody. Beads were washed, and the protein eluted into loading buffer and analysed via immunoblot.

### 2.7 Chromatin immunoprecipitation (ChIP)

Method was performed on LNCaP and 22Rv1 cells as well as on human tissue [47]. Cell lines, seeded into 15 cm plates at 5 × 10^6^ cells per plate, grown in PRF‐RPMI + 10% DCC‐FCS were treated with either 10 nm DHT or an equivalent volume of vehicle (ethanol), for 4 h before fixation with 1% formaldehyde. For tissue, samples were cut into cubes no bigger than 0.3 cm^3^ before fixation, emulsified into single cell solutions and sonicated as described previously [48, 49]. After lysis and sonication, samples were incubated with 5 ng per sample of antibodies against either AR (SantaCruz, sc‐816x, sc‐7305x), TRIM24 (SantaCruz, sc‐271266), or TRIM28 (Invitrogen, 730029), with IgG control (Santa Cruz, sc‐2025). For ChIP with siRNA, C4‐2B and 22Rv1 cells were seeded into 15 cm plates and grown in RPMI + 10% FCS, transfected with 5 μm of siRNA, and ChIP performed after 3 days.

Publicly available ChIP‐seq data for H3K27ac: GSM686937 (GSE27823), GSM1249448 (GSE51621); AR: GSM2480801 (GSE94682), GSM3148986 (GSE114732), GSM1868866 (GSE72714); TRIM24: (GSM1697902 (GSE69331), GSM2891162 (GSE108144)), TRIM28: (GSM2480827 (GSE94682) and GSM2891164 (GSE108144)) were analysed via Cistrome analysis pipeline [36, 37, 50, 51]; Snakemake analysis pipeline, read mapping/filtering/sorting using bwa then samtools, peaks called with MACS2 (*q* < 0.01). Data were visualised using WashU Epigenome Browser [51]. Galaxy DiffBind tool was used for differential peak analysis with deseq2 [52]. Genes associated with AR, TRIM24 and TRIM28 data were assessed using BETA‐minus analysis using Cistrome [38].

### 2.8 ChIP‐reChIP

ChIP‐reChIP was performed in LNCaP, C4‐2B and 22Rv1 cells as per previous published methods [53] with amendments. In brief, cells were treated with 10 nm DHT or an equivalent volume vehicle (VC) for 4 h, then to maximise nuclear receptor–chromatin binding, cells were fixed in a solution of PBS, 1 mm MgCl_2_, 1 mm CaCl_2_ and 2 mm disuccinimidyl‐glutarate prior to formalin fixation [49]. Furthermore, to enhance protein pulldown, human control RNA (4307281, Thermo Fisher) and recombinant histone 2B (M2505S, New England Biolabs) were added to the immunoprecipitation steps [48, 49].

### 2.9 Immunofluorescence (IF)

AR, TRIM24 and TRIM28 were visualised in LNCaP and 22RV1 cells via immunofluorescence methods previously described [54]. Briefly, cells seeded onto glass coverslips in 24 well plates (1 × 10^5^ cells per well) were treated with combinations of 10 nm DHT, 1 μm of Enzalutamide or equivalent vehicle control. Cells underwent fixation with 1% formaldehyde before PBS‐T washes, blocking with BSA and overnight incubation with primary antibodies (anti‐AR 1/5000, anti‐TRIM24 1/1000, anti‐TRIM28 1/1000). Primary antibodies were visualised using Alexa Fluor^®^ 488 goat anti‐mouse IgG (A‐11029, Molecular Probes, Life Technologies, Eugene, OR, USA) or Alexa Fluor^®^ 568 goat anti‐rabbit IgG (A‐11036, Molecular Probes, Life Technologies). Nuclei were stained using DAPI. Fluorescence was captured using an EVOS microscope (Thermo Fisher).

### 2.10 Immunohistochemistry (IHC)

A tissue microarray (TMA) was previously created from 99 PCa patients, using samples from the Imperial College Healthcare Tissue Bank (ICHTB). ICHTB is supported by the National Institute for Health Research (NIHR) Biomedical Research Centre based at Imperial College Healthcare NHS Trust and Imperial College London. ICHTB is approved by Wales REC3 to release human material for research (22/WA/0214), and the samples for this project (R14122). From each patient, 2 cores from pathologist‐defined regions of cancer and benign tissue were included [55]. Fixed material from PDE samples also underwent IHC analysis.

Immunohistochemistry method was adapted from previous studies [28, 56]. Heat induced antigen retrieval was used with 0.01 m citrate buffer solution (pH 6.0). Sections were washed in PBS before incubating ON with antibodies against TRIM24, TRIM28 and VEGF (as used in immunoblot section). Bound antibodies were detected using Histostain‐Plus IHC HRP Kit and DAB+ (Life Technologies).

Staining was assessed independently by two scientists, blinded, on a scale of staining intensity ranging from 0 (no staining), 1+, 2+, 3+ (strong). Scores were averaged for each sample. Average staining score was assessed according to clinical characteristics or correlations using IBM spss Statistics 24.

### 2.11 Tube formation assays

The ability of HUVEC cells to form tubes in the presence of conditioned media from cancer cells transfected with siRNA against TRIM proteins was assessed using the Abcam kit (ab204726). Briefly, 96‐well plates were coated with 50 μL of Matrigel which was allowed to set for 1 h before applying 1 × 10^4^ HUVEC cells per well in 50 μL of media. Added to each well was 50 μL of CM from LNCaP, C4‐2B and 22RV1 cells transfected with siRNA as above. Media containing 10 ng·mL^−1^ of VEGF was used as a control. HUVECs were then incubated, and photographs were taken after 6‐ and 18‐h incubation. Tube formation was assessed manually by counting the number of ring structures formed and the number of branching points. Tube formation was also assessed using the Angiogenesis macro in fiji [57, 58].

### 2.12 Conditioned media

Conditioned media was collected after fresh media was incubated with the cells for 48 h or from explants for 72 h, media was collected, centrifuged at high speed for 5 min and filtered (0.22 μm) before use.

### 2.13 LDH assay

To detect LDH released from cells as a marker of cell death, we utilised the LDH‐Glo Cytotoxicity Assay (J2381, Promega, Madison, WI, USA). Assays were completed using conditioned media from explant and cell culture experiments. Assay was performed as per the manufacturer's instructions.

### 2.14 VEGF ELISA

Secreted VEGFA was detected with the Human VEGF‐A ELISA Kit (BMS277‐2, Invitrogen). The assay was performed on conditioned media from explant models and cell lines as per the manufacturer's instructions.

## 3 Results

### 3.1 Expression levels of the bromodomain‐containing coregulators TRIM24 and TRIM28 are upregulated in CRPC

**Fig. 1:**
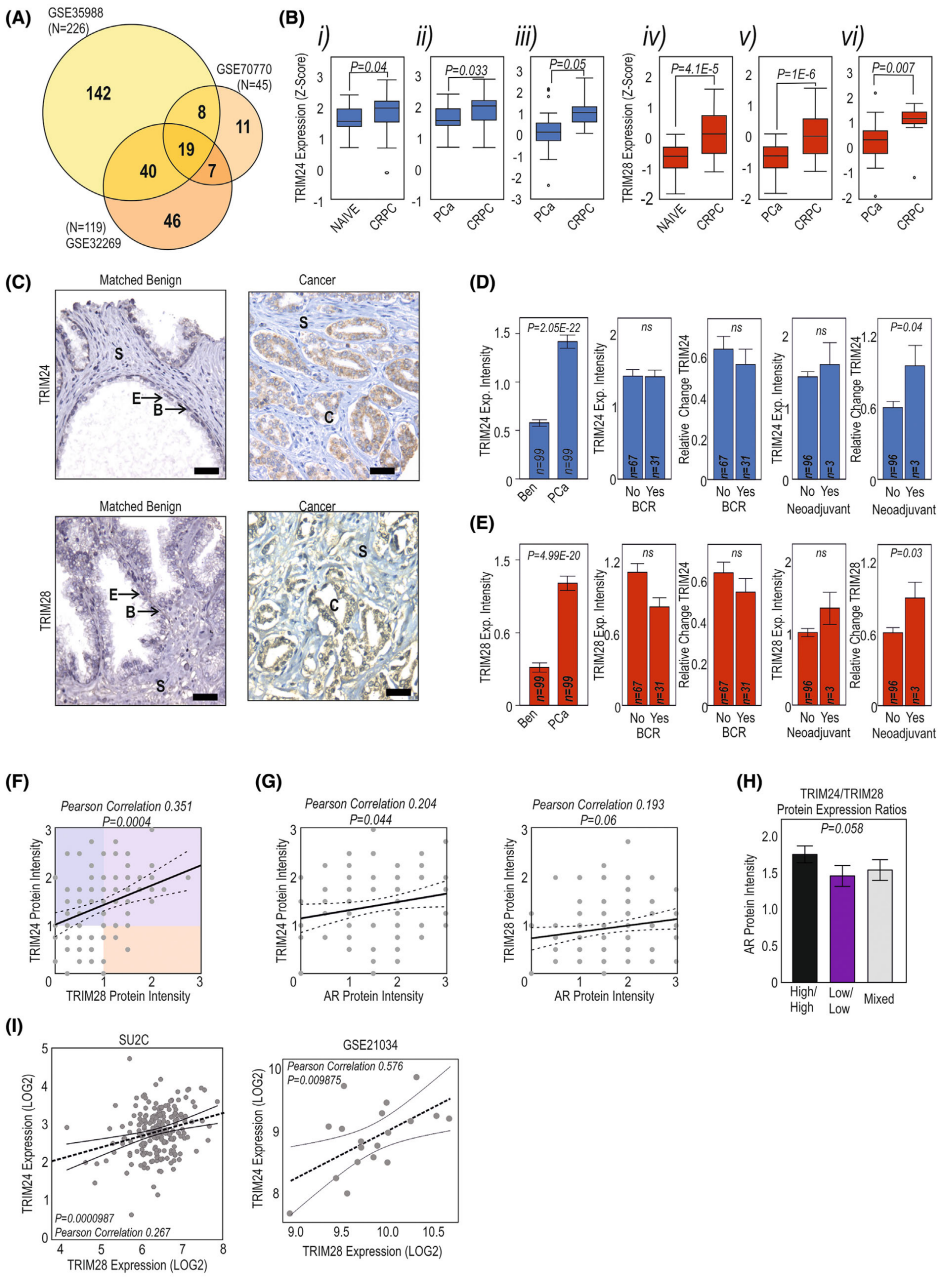
TRIM24 and TRIM28 expression increases with disease progression (A) Expression analysis of coregulators significantly altered (*P* < 0.05, LFC > 0.5) in castrate‐resistant prostate cancer (CRPC) compared with non‐CRPC/primary prostate cancer (PCa) samples across three different data sets (GSE35988, GSE70770 and GSE32269). Box plots of median expression across GSE35988 naïve *n* = 59, CRPC *n* = 35; GSE70770 PCa *n* = 23, CRPC *n* = 8, GSE32269 PCa *n* = 22, CRPC *n* = 29. (B) Expression analysis of TRIM24 (i–iii) and TRIM28 (iv–vi) in CRPC samples (i/iv) GSE32269 (CRPC compared to primary PCa, hormone treatment ‘Naïve’ samples), (ii/v) GSE35988 (CRPC compared to localised PCa), (iii/vi) GSE70770 (CRPC compared to localised PCa samples). Significance determined via adjusted *P*‐value (Benjamini–Hochberg). (C) Examples of immunohistochemical staining of TRIM24 or TRIM28 in a tissue microarray (TMA) of 99 patients with cores from both cancer and matched benign tissue from each patient. B, basal cells; C, cancer; E, epithelial cells; S, stroma. Scale bar = 25 μm. Expression intensity scores for (D) TRIM24 and (E) TRIM28 were compared between prostate cancer (PCa *n* = 99) samples and matched benign samples (Ben *n* = 99), future biochemical relapse (BCR), and patients who did vs. did not receive neoadjuvant hormonal therapy. TRIM24 and TRIM28 expression was evaluated in all these settings, and as a mean change between the cancer samples and their adjacent normal tissue. Data presented as mean of *n* = 99 with SEM. Significance determined via *t*‐test. (F) Correlation between TRIM24 and TRIM28 staining intensities in *n* = 99 PCa samples. (G) Correlation of TRIM24 and TRIM28 protein expression with AR expression in TMA cohort (*n* = 99). Correlation measure with Pearson method. (H) Protein expression of AR in TMA samples grouped according to expression of TRIM proteins, High TRIM24 and TRIM28 (High/High, *n* = 35) or Low TRIM24 and TRIM28 (Low/Low, 21), or other combinations of TRIM expressions (Mixed, *n* = 44). Data represent mean ± SEM. Significance determined via *t*‐test. (I) Correlation of TRIM24 and TRIM28 mRNA in metastatic PCa samples from the SU2C (left, *N* = 209) and GSE21034 (right, *n* = 37) cohort. Pearson correlation was used to analyse relationships in expression.

Analysis of the over 300 known nuclear receptor (NR) coregulators across three publicly available datasets identified those differentially expressed in CRPC, of which 19 were consistently differentially expressed in all three databases (Fig. 1A, Table S2). These notably included upregulation of two of the subgroup of bromodomain‐containing TRIM family members, *TRIM24* and *TRIM28* (Fig. 1B). Expression of the third member of this subgroup, *TRIM33*, was not increased in CRPC (Fig. S1A). Expression of both *TRIM24* and *TRIM28* was strongly associated with AR expression (Fig. S1B). We further analysed *TRIM24*/*28* expression data from 259 samples taken from different metastatic disease sites (GSE6919) and found significantly increased expression of *TRIM24* at all metastatic sites compared to primary tumours, while *TRIM28* was also increased in liver metastases and at metastatic sites other than lymph, bone and other soft tissue (which includes adrenal and muscle) (Fig. S1C).

We investigated whether this higher expression was evident also at protein levels in a tissue cohort which includes both cancer and matched benign tissue from 99 patients (examples in Fig. 1C). In benign tissue, TRIM24 and TRIM28 expression was minimal or absent in the luminal epithelial cells, but present in basal epithelial cells. The intensity levels of both TRIM24 and TRIM28 were significantly higher in cancer tissue than in the matched benign tissue (Fig. 1D,E, *P* < 0.001, *P* < 0.05), although there was no significant difference between individual Gleason grades (Fig. S1Di). There was no significant difference in TRIM24 and TRIM28 protein levels between patients who later relapse (biochemical relapse, BCR) and those who do not, or in the relative change in TRIM24 or TRIM28 expression in the cancer samples compared to their own benign tissue (Fig. 1D,E). In cancer samples from patients that had received neoadjuvant hormone therapy, overall, the change in TRIM24 and TRIM28 expression between cancer and benign tissue was significantly increased compared to those who did not receive this, although numbers are small (Fig. 1D,E, *P* = 0.04 and *P* = 0.03, respectively). Additionally, the expression and change in expression between cancer tissue and the patients' own benign tissue of TRIM24 and TRIM28 were significantly correlated to each other in these patient tissues (Fig. 1F, *P* < 0.001, Fig. S1Dii). Both TRIM24 and TRIM28 expression correlated with AR expression in cancer tissue (Fig. 1G), and patient tissue with high TRIM24 and TRIM28 protein expression also demonstrated a trend (nonsignificant) toward higher expression of AR (Fig. 1H). We also find expression correlation between the two TRIM genes in two metastatic cohorts [59, 60](Fig. 1I).

In summary, in primary tissue, TRIM24 and TRIM28 RNA and protein expression levels are correlated, increased in cancer, but are not indicative of future relapse. However, both TRIM24 and TRIM28 expression increased in advanced and metastatic disease and were associated with CRPC, which is perhaps why there was an association between neoadjuvant therapy and increased expression in the primary site. Combined, these data suggest that while increased between cancer and normal/benign tissue, even between normal/benign and Gleason 6 tumours, TRIM24 and TRIM28 may also be of importance in advanced disease progression and CRPC.

### 3.2 TRIM24 and TRIM28 expression and interaction with AR

**Fig. 2:**
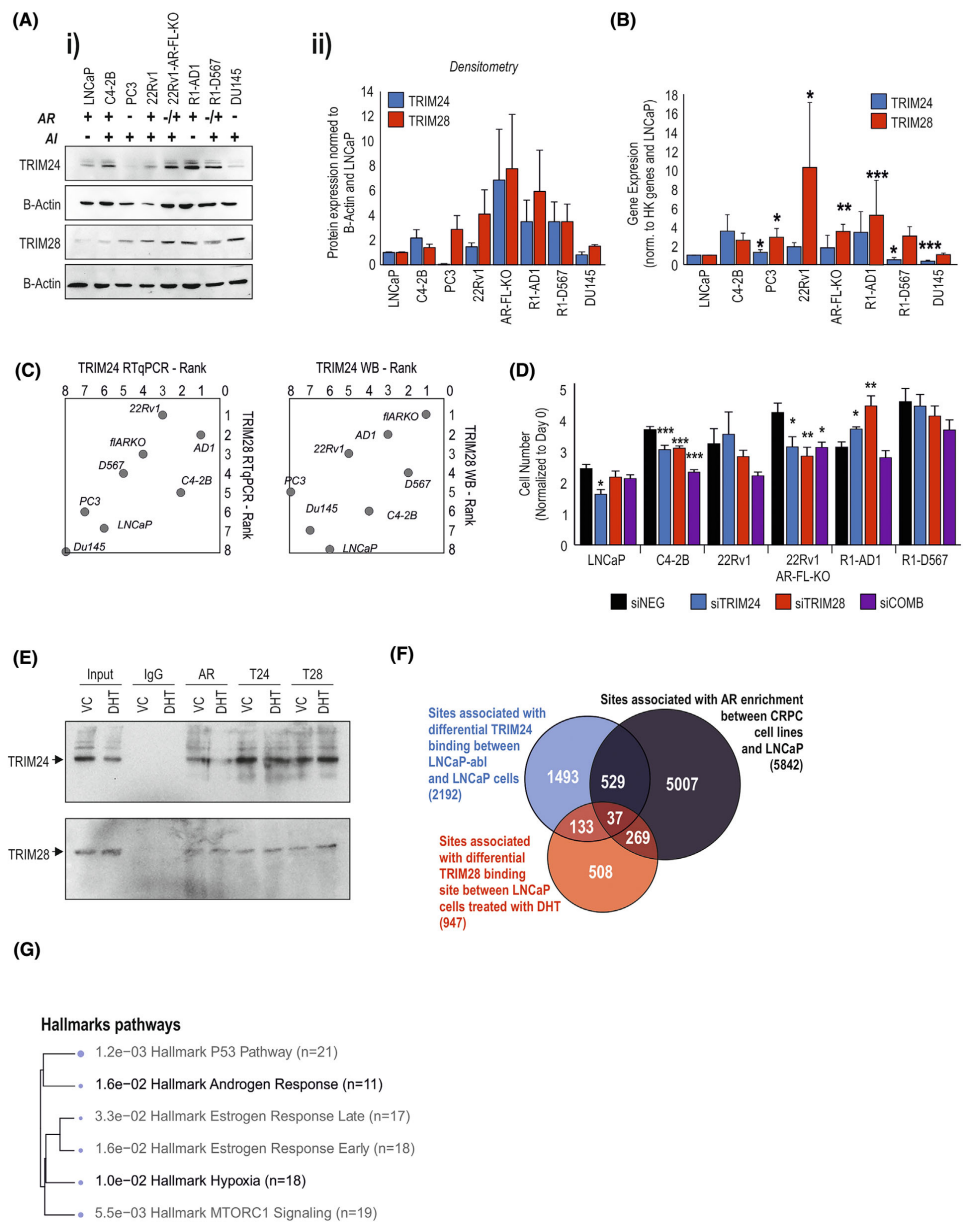
TRIM24 and TRIM28 interactions with androgen receptor (AR). (A) (i) Immunoblot of TRIM24 and TRIM28 expression across cell lines, separated into AR‐positive/negative and androgen independence (AI), with (ii) densitometry across samples, normalised to the B‐Actin control of each sample (mean of *n* = 3 ±SD). (B) TRIM24 and TRIM28 mRNA expression in each cell line (mean of *n* = 3 ±SD). Significance determined by Student *t*‐test, *, **, *** = *P* < 0.05, 0.01, 0.001, compared to LNCaP (set at 1). (C) Comparison ranking of cell lines for TRIM24 and TRIM28 mRNA and protein expression. Data presented as mean of *n* = 3. (D) Crystal violet proliferation assay of LNCaP, C4‐2B, 22Rv1, 22Rv1 AR‐FL‐KO, R1‐AD1 and R1‐D567 cells transfected with siRNA against TRIM24 or TRIM28 alone or in combination. Time point is 7‐day post‐transfection, data represent mean *n* = 4 biological repeats ±ECM. Significance compared to siNEG control via Student *t*‐test = *P < 0.05, **P < 0.01, *** = *P* < 0.001. (E) Immunoblots from Co‐immunoprecipitation (CoIP) lysates of 22rv1 cells treated with 10 nm dihydrotestosterone (DHT) or equivalent vehicle control (VC) and measuring endogenous protein interactions. Representative of 2 repeats CoIP, immunoprecipitation with IgG control, AR, TRIM24 or TRIM28 as indicated, then immunoblotting for TRIM24 and TRIM28 protein. (F) Venn diagram of the overlap between genes associated with TRIM24, TRIM28 and AR binding sites (BS). Data are from chromatin immunoprecipitation (ChIP) experiments probing AR between castrate‐resistant prostate cancer (CRPC) cell lines and androgen‐sensitive LNCaPs [22, 33, 34, 35], and ChIPs for TRIM24 and TRIM28 in LNCaP cells [22, 25, 111]. (G) Pathway analysis of genes associated with binding sites of AR, TRIM24 and TRIM28.

To model the role of these TRIM proteins in CRPC, we assessed their expression across several PCa cell lines that differ in AR expression and responsiveness to/dependence on androgens. Compared to the AR‐positive, androgen‐dependent line LNCaP: at a protein level, TRIM24 was more highly expressed in androgen‐insensitive cell lines that express some form of AR (C4‐2B, 22Rv1 and derivative AR‐FL‐KO, R1‐AD1 and derivative R1‐D567) and was very low in AR‐null cell lines (PC3, DU145), while TRIM28 was lowest in LNCaP and C4‐2B cells, and relatively consistent (and higher) across the androgen‐insensitive cell lines (Fig. 2A,C and Fig. S2A). At an RNA level, *TRIM24* was highly expressed in C4‐2B, R1‐AD1 and 22Rv1 models, *TRIM28* was most highly expressed in 22Rv1 cells (Fig. 2B,C and Fig. S2A). Proliferation assays in multiple PCa cell lines transfected with siRNA against *TRIM24*, *TRIM28*, or a combination of the two (Fig. S2B,C) showed (using two different assays) that knockdown was able to reduce proliferation in most cases (though increased in R1‐AD1) to varying degrees with little relation to AR status or androgen sensitivity (Fig. 2D and Fig. S2D).

To investigate the relationship between TRIM24, TRIM28 and continued AR activity in CRPC, we assessed first their physical interaction with AR. In experiments assessing ectopically expressed proteins, members of the TRIM24, TRIM28 and TRIM33 subfamily form heterodimers [13, 61]. TRIM28 is also able to stabilise TRIM24 expression via interaction with SPOP [25]. Assessment of endogenous protein interactions in 22Rv1 cells via CoIP identified that native TRIM24 and TRIM28 proteins physically interact and that both interact with AR (Fig. 2E). However, the other bromodomain member of the TRIM family, TRIM33, is noted to interact with TRIM24 or TRIM28 [13] but we were unable to detect this interaction.

TRIM24 and TRIM28 are known to regulate AR activity [16, 18, 21, 22, 25]; using publicly available sequencing data from prostate cancer cells, we assessed sites which had changes in chromatin state between CRPC model cell lines and androgen‐sensitive cells, TRIM24 binding patterns in androgen‐insensitive LNCaP compared to sensitive LNCaP cells, and differential TRIM28 binding patterns in LNCaPs in response to androgens (Fig. 2F). We assessed genes associated with these changes for AR, TRIM24 and TRIM28, finding 566 genes associated with both AR and TRIM24, and 306 genes associated with TRIM28 and AR (Fig. 2F). Pathway analysis of these genes potentially associated with AR and either TRIM24 or TRIM28 supports roles in hormone signalling, cancer, P53 and mTOR signalling, documented areas of coregulator influence on AR signalling (Fig. 2G). Some of the more novel pathways identified potential roles in angiogenesis/hypoxia signalling (Fig. 2G).

### 3.3 TRIM24 and TRIM28 influence androgen regulation of target genes

**Fig. 3:**
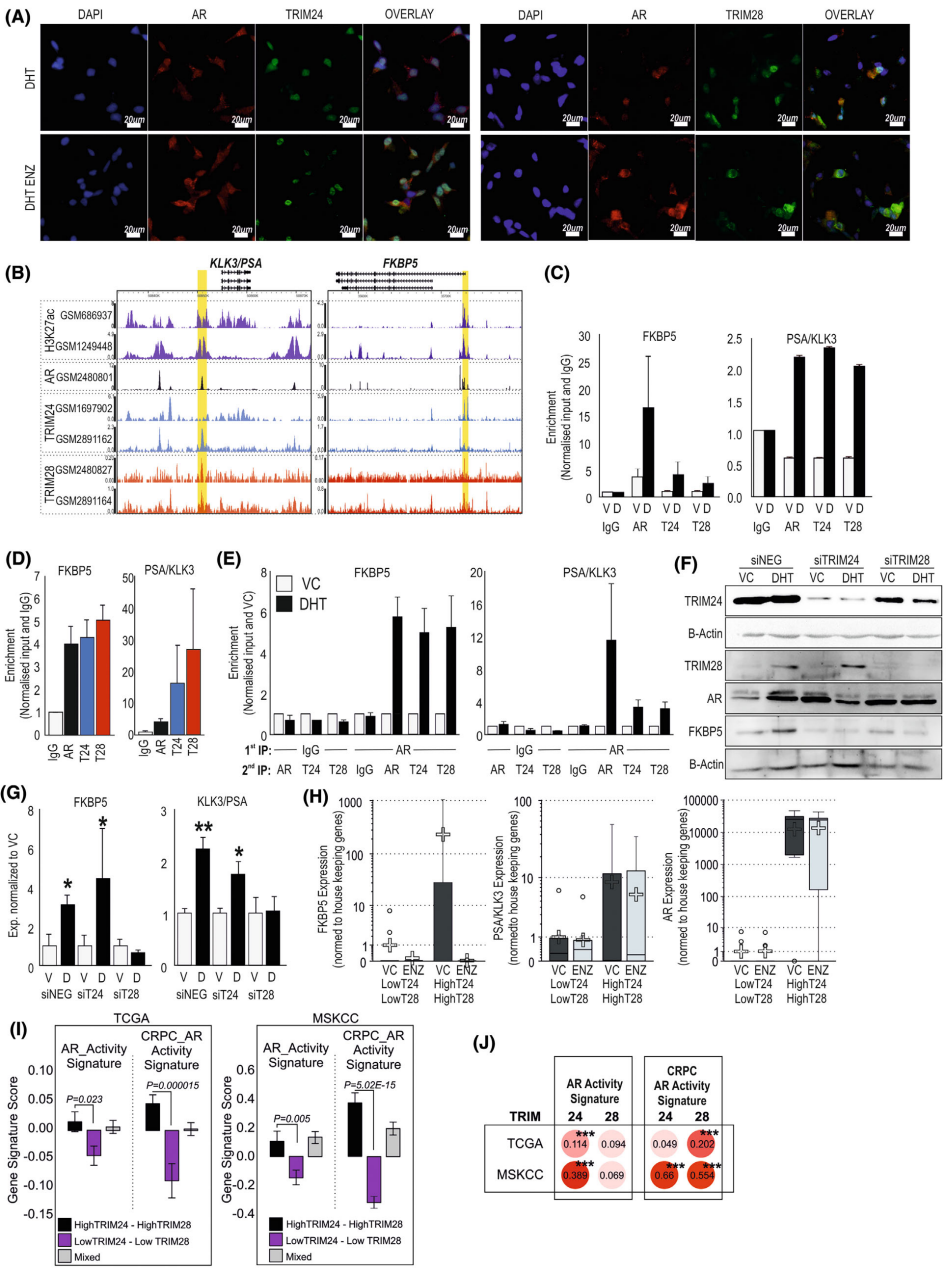
Interactions between TRIM24, TRIM28 and androgen receptor (AR) with each other and chromatin. (A) Immunofluorescent localisation of TRIM24, TRIM28 and AR in response to dihydrotestosterone (DHT) ± Enzalutamide (ENZ). LNCaP cells were treated with 10 nm DHT alone or in combination with 10 μm ENZ, for 1 h before fixation and immunofluorescent staining. Nucleus is stained blue with DAPI, AR is red, and either TRIM24 or TRIM28 (as labelled) is green. Representative image of *n* = 3 experiments. Scale bar = 20 μm (B) Example of H3K27ac (GSM686937 [112], GSM1249448 [113]), AR (GSM2480801 [111]), TRIM24 (GSM1697902 [22], GSM2891162 [25]), TRIM28 (GSM2480827 [111], GSM2891164, [25]) binding in ChIP‐seq experiments completed in LNCaP cells. Yellow area indicates region primers for ChIP‐qPCR were designed for. (C) ChIP‐qPCR of candidate genes in LNCaP cells treated with 10 nm DHT or equivalent vehicle control (VC). Immunoprecipitation (IP) was performed with AR, IgG, TRIM24 or TRIM28 and PCR for target regions in genes as indicated. Data are representative of five biological repeats normalised to input and IgG, with error bars representing SEM. (D) RT‐qPCR from ChIP of AR, TRIM24, TRIM28 completed in four prostate cancer samples (mean of *n* = 4 ± SEM shown) normalised to input and IgG. (E) Results from ChIP‐reChIP in LNCaP cells treated with 10 nm DHT or equivalent VC, where first immunoprecipitation (IP) was performed with either anti‐AR antibody or IgG, then a second IP for AR, TRIM24, TRIM28 or IgG. Data represented mean of *n* = 5 ± SD. (F) Immunoblot analysis for LNCaP cells transfected with siRNA then treated with 10 nm DHT or vehicle control (VC) for 48 h. Protein levels of TRIM24, TRIM28, AR and FKBP5 were measured with B‐Actin as loading control (B‐Actin under relevant proteins on same blot). Representative image of *n* = 3 biological repeats. (G) RT‐qPCR analysis of AR‐TRIM target genes in LNCaP cells transfected with siRNA against TRIM24 and TRIM28 or scr‐control and treated with 10 nm DHT (D) or equivalent vehicle (V). Data represent mean of *n* = 3 ± SD. Significance determined via Student *t*‐test comparing DHT treatment to VC, *P < 0.05, **P < 0.01. (H) Analysis of target genes in PDE of PCa patient tissue treated with 10 μm ENZ or equivalent vehicle control (VC) for 3 days. Sample were grouped based on high or low expression of bother TRIM24 and TRIM28 genes (*N* = 9 for each subgroup). Expression of mRNA of AR and target genes was assessed, represented as box plots, statistics calculated with Kruskal–Wallis. With means shown as crosses. (I) Expression of AR activity gene signatures and CRPC‐AR gene signature in TCGA and MSKCC data cohorts. Patient samples grouped according to expression levels of TRIM24 and TRIM28. Independent *t*‐test used to determine significance between groups. (J) Person correlation of TRIM24 and TRIM28 with the two gene signatures in the TCGA and MSKCC datasets. ***P < 0.001.

Both TRIM24 and TRIM28 are well noted for their roles in coregulating AR activity, so we next wanted to confirm the functional consequences of these interactions within our models. Immunofluorescent staining confirmed mostly nuclear location of AR and TRIMs in the presence of DHT, while in the presence of enzalutamide, AR is partially detectable in the cytoplasm, but both TRIM24 and TRIM28 remain in the nucleus (Fig. 3A). Analysis of ChIP datasets confirms that in the nucleus, AR and TRIM24 and TRIM28 interact with open chromatin regions (H3K27ac peaks) associated with classical ARGs, KLK3 and FKBP5 (Fig. 3B). We confirmed this with ChIP experiments undertaken in cell lines (Fig. 3C) and patient PCa tissue (Fig. 3D). To determine whether the AR and TRIM proteins bind simultaneously, we performed ChIP‐reChIP using an initial IP with anti‐AR antibody, then a second IP for either TRIM24 or TRIM28. Both TRIM24 and TRIM28 were detectable at AR‐bound chromatin regulatory regions associated with *PSA/KLK3* and *FKBP5* genes (Fig. 3E), confirming that they are recruited simultaneously with AR to these regions. Silencing TRIM24 or TRIM28, or silencing both TRIMs reduced the binding of AR, and silencing either TRIM partially reduced the binding of the other TRIM protein (Fig. S2E). FKBP5 displayed reduced or complete abrogation of DHT‐stimulated upregulation at the protein level when either TRIM24 or TRIM28 were silenced in androgen‐sensitive LNCaP cells, though less so at the RNA level (Fig. 3F,G). *KLK3*/*PSA* androgen sensitivity was reduced at the RNA level (Fig. 3G). In PDEs from PCa patients, we find that the expression of AR and AR target genes is higher in those patients which express high levels of TRIM24 and TRIM28 (Fig. 3H) and that changes in response to enzalutamide are able to alter the expression of the genes (Fig. 3H). Furthermore, in TCGA and MSKCC patient cohorts, high expression of both *TRIM24* and *TRIM28* is associated with increased expression of an AR activity signature [41] and CRPC‐AR activity signature [42] (Fig. 3I). Both *TRIM24* and *TRIM28* tended to have a strong correlation with the CRPC‐AR activity signature (Fig. 3J).

For the first time, we show TRIM24 and TRIM28 chromatin interactions in patient tissue, and overall, these data indicate that AR, TRIM24 and TRIM28 interact at the chromatin level to regulate target genes. Specifically, TRIM24 and TRIM28 are affecting AR signalling in PCa, potentially regulating gene targets involved in aiding continual growth/survival, leading us to hypothesise that targeting both these TRIMs in combination with AR may reduce tumours by preventing proliferation.

### 3.4 TRIM24 and TRIM28 influence proliferation and alter response of therapy‐resistant prostate cancer cells to enzalutamide

We next wanted to investigate how TRIM24 and TRIM28 affect the response of cancer cells to antiandrogen therapies. In androgen/antiandrogen‐sensitive cells, silencing TRIM24 and TRIM28 alone or in combination reduced the response to DHT and the response to antiandrogens in R1‐AD1 [27] and only when in the combination in LNCaP cells (Fig. S3A). The proliferation driver *MYC* is a known androgen‐regulated gene, and we show that both TRIM24 and TRIM28 bind to the regulatory regions of MYC with AR (Fig. S3B–E); silencing either TRIM24 or TRIM28 abolished androgen upregulation of MYC RNA/protein (Fig. S3F,G).

**Fig. 4:**
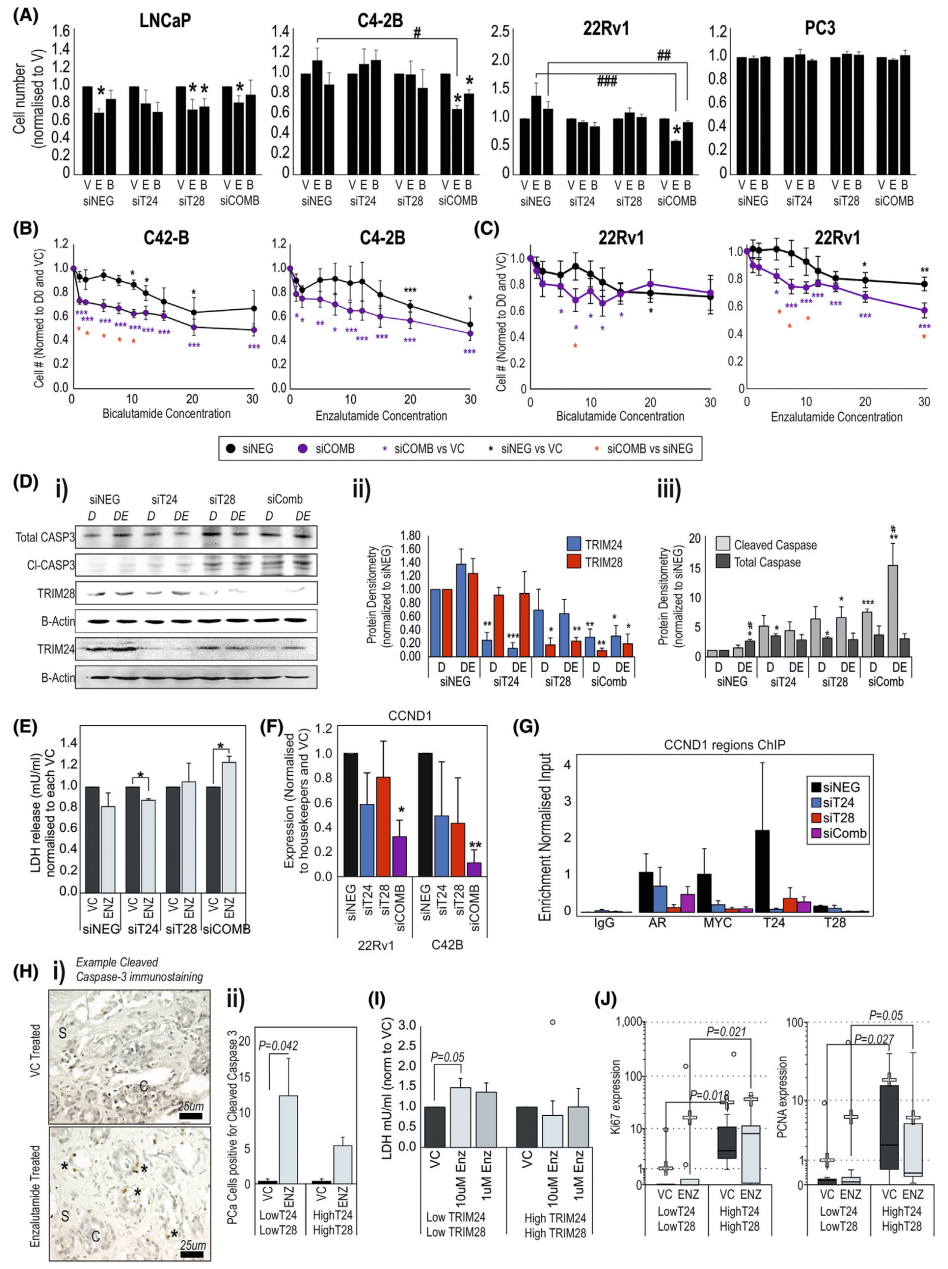
Expression of TRIM24 and TRIM28 influences response of prostate cancer (PCa) to antiandrogens. (A) Crystal violet proliferation assays measuring the response of LNCaP, C4‐2B, 22Rv1 and PC3 cells transfected with siRNA against TRIM24 (siT24) or TRIM28 (siT28) alone or in combination, or a negative control, then treated with 10 μm Enzalutamide (E) or Bicalutamide (B) compared to vehicle control (V). Cell number measured after 6 days, presented as *N* = 4 ± SEM. Significance determined by Student *t*‐test, * = *P* < 0.05 E or B compared to V for each siRNA condition. #, ## and ## = *P* < 0.05, 0.01 and 0.001 compared to the same treatment for siNEG condition. Dose response of (B) C4‐2B and (C) 22Rv1 cells transfected with siRNA against both TRIM24 and TRIM28 (siCOMB) to increasing concentrations of bicalutamide and enzalutamide [μm], compared to C4‐2B cells transfected with siRNA control (siNEG). Data represent mean of *n* = 4 ± SEM. Student *t*‐test comparing dose of enzalutamide of bicalutamide to VC in siNEG cells (Black asterisks) or siCOMB (purple asterisks), also comparison between siNEG and siCOMB (red asterisks). * = *P* < 0.05, ** = *P* < 0.01, *** = *P* < 0.001. (D) (i) Immunoblot analysis of 22Rv1 cells transfected with siRNA against TRIM24 and/or TRIM28, or a scrambled control, treated with 10 nm dihydrotestosterone (DHT) alone (D) or with 5 μm Enzalutamide (DE) for 48 h. B‐Actin control is under images of proteins on the same blot. (ii) Densitometry of TRIM24 and TRIM28, and (iii) cleaved and total caspase in transfected cells (mean ± SD of *n* = 3). Significance determined by Student *t*‐test between siNEG controls and cells transfected with siRNA, * = *P* < 0.05, **P < 0.01, *** = *P* < 0.001, and between DE and D samples # = *P* < 0.05. (E) LDH assay detecting secreted LDH in conditioned media as a marker of cell death. Data represent LDH concentration detected in conditioned media from cell lines transfected with siRNA against TRIM24, TRIM28 or both then treated with enzalutamide or control. Data represent mean of three biological replicates and is normalised to the VC‐treated conditioned media. Error bars indicate SD. Significance determined with Student *t*‐test, * = *P* < 0.05. (F) CCND1 expression in C4‐2B and 22Rv1 cells transfected with siRNA against TRIM24 and TRIM28 before treatment with 10 μm enzalutamide. Data represent the mean of three biological replicates, normalised to the negative control samples, error bars = SD. Significant between enzalutamide and VC‐treated samples determined with Student *t*‐test * = *P* < 0.05, ** = *P* < 0.01. (G) ChIP experiment depicting binding of AR, MYC, TRIM24 and TRIM28 to the regulator region of CCND1 in C4‐2B cells transfected with siRNA against TRIM24 and or TRIM28. Enrichment determined relative to Input control. Data represent mean from three biological repeats, error bars = SD. (H) Patient‐derived explant (PDE) models, where patient PCa tissue has been cultured *ex‐vivo* for 72 h in the presence of 10 μm Enzalutamide or equivalent vehicle control (VC). Scale bar = 25 μm. (i) Examples of PCa PDE tissue stained for cleaved caspase 3 after treatment with either enzalutamide or equivalent VC. Examples of cancer designated with ‘C’, stroma with ‘S’, cleaved caspase with ‘*’. (ii) Mean number of cleaved caspase 3‐positive cells per field of view per sample with a minimum 100 PCa cells counted. Samples with low TRIM24 and TRIM28 expression *n* = 8, high expression of TRIM24 and TRIM28 *n* = 9. Error bars indicate SEM. Significance determined with Student *t*‐test. (I) LDH assay detecting secreted LDH in conditioned media as a marker of cell death. Data represent LDH concentration detected in conditioned media from explant models, normalised to conditioned media from VC‐treated explant tissue of each patient (low TRIM24 and TRIM28 expression *n* = 8, high expression of TRIM24 and TRIM28 *n* = 9). Error bars indicate SEM. Significance determined with Student *t*‐test. (J) RT‐qPCR expression analysis of proliferation markers in patient‐derived explant (PDE) samples after enzalutamide treatment. Samples grouped based on either high TRIM24 and TRIM28 expression (*n* = 9) or low expression (*n* = 10), samples with mixed TRIM expression excluded. Displayed are medians within box plots, with means displayed as crosses. Significance determined via Mann–Whitney *U*‐test.

In the cell lines that express only AR variants and not the full‐length AR (22Rv1‐AR‐FL‐KO and R1‐D567 cells), these cells were resistant to enzalutamide regardless of the absence of TRIM proteins (Fig. S4A), presumably due to the lack of the AR ligand‐binding domain. In the AR‐negative PC3 cells, there was no response to enzalutamide or bicalutamide, as expected, regardless of TRIM silencing (Fig. 4A). Intriguingly, in AR‐positive androgen‐insensitive cells (C4‐2B and 22Rv1), when both TRIM24 and TRIM28 are silenced, and only together, C4‐2B and 22Rv1 cells become sensitive to 10 μm enzalutamide, and C4‐2B cells also to Bicalutamide (Fig. 4A and Fig. S4A,B), to a similar degree of response to these antiandrogens as seen in LNCaP cells. We explored this further with a dose scaling and showed that this response is consistent, even at low doses. The combination of silencing TRIM24 and TRIM28 made C4‐2Bs sensitive to concentrations as low as 1 μm of bicalutamide and enzalutamide compared to 10 and 20 μm, respectively, in siNEG (Fig. 4B) and 22Rv1 cell sensitive to concentrations as low as 5 μm of bicalutamide and enzalutamide compared to 20 μm of either in siNEG cells (Fig. 4C). We investigated further in 22Rv1 cells and saw that the siRNA‐mediated reduction in TRIM28 induced a cleaved caspase 3 response to enzalutamide, and that this response was then even greater when both TRIM24 and TRIM28 were silenced, suggestive of a role for TRIM24 and TRIM28 mediating apoptotic responses to enzalutamide (Fig. 4D). In conditioned media, secreted LDH as a marker of cell death was increased in response to enzalutamide only when both TRIM24 and TRIM28 were silenced (Fig. 4E). In both C4‐2B and 22Rv1 cells, cyclin D1 (*CCND1*) a cell cycle regulator and target of both AR and MYC, is reduced when both TRIM24 and TRIM28 are silenced (Fig. 4F). Furthermore, the binding of AR and MYC to cyclin d1 regulator regions (was reduced when both TRIM24 and TRIM28 were silenced (Fig. 4G)). In PDE models, when we assessed cleaved caspase‐3 levels in response to enzalutamide, the number of positive cells in response to enzalutamide was higher in cells with lower levels of both TRIM24 and TRIM28 (Fig. 4H). In the conditioned media of these explant samples, detection of secreted LDH was significantly increased in response to enzalutamide in samples expressing low TRIM24 and TRIM28 (Fig. 4I). In addition, at an RNA level, the proliferation markers *Ki67* and *PCNA* are increased in the PDEs with higher levels of *TRIM24* and *TRIM28* (Fig. 4J).

Supporting the above findings of TRIM24 and TRIM28 involvement in growth, in an *in vivo* model of PCa hyperplasia (driven by *Pten* deletion [62]), both *Trim24* and *Trim28* expression is markedly increased in the prostates of *Pten* homozygous deletion mice compared to either the heterozygous (no phenotype) or wild‐type *Pten* littermates (Fig. S4C), supporting the association with hyper/unchecked proliferation in the prostate. Treatment of mice with enzalutamide significantly increased *Trim24* (*P* < 0.01, similar to what was seen in clinical data with neoadjuvant therapy) but not *Trim28* expression (Fig. S4D). A bromodomain inhibitor, IBET (designed to inhibit Brd4 but shown to also inhibit other bromodomain‐containing proteins including TRIM24/28 [15, 63, 64]), could also reduce *Trim24* and *Trim28* expression and proliferative markers in these mice (Fig. S4D). Cell line data suggest that some of the effects of bromodomain inhibitors could work through TRIM24 and TRIM28 as the response to JQ1 and IBET is diminished when either TRIM24 or TRIM28 is silenced (Fig. S4E).

Together, these data suggest that TRIM24 and TRIM28 may have a role in regulating the response of androgen‐insensitive cells to enzalutamide and bicalutamide, reflective of CRPC, and that targeting them may resensitise cancer cells to antiandrogens. Strikingly, in our cell line models showing no apoptotic response to enzalutamide, knocking down TRIMs 24 and 28 simultaneously led to a marked apoptotic response to enzalutamide treatment.

### 3.5 TRIM24 and TRIM28 expression associates with VEGFA, endothelial markers and vascular invasion in patient samples

**Fig. 5:**
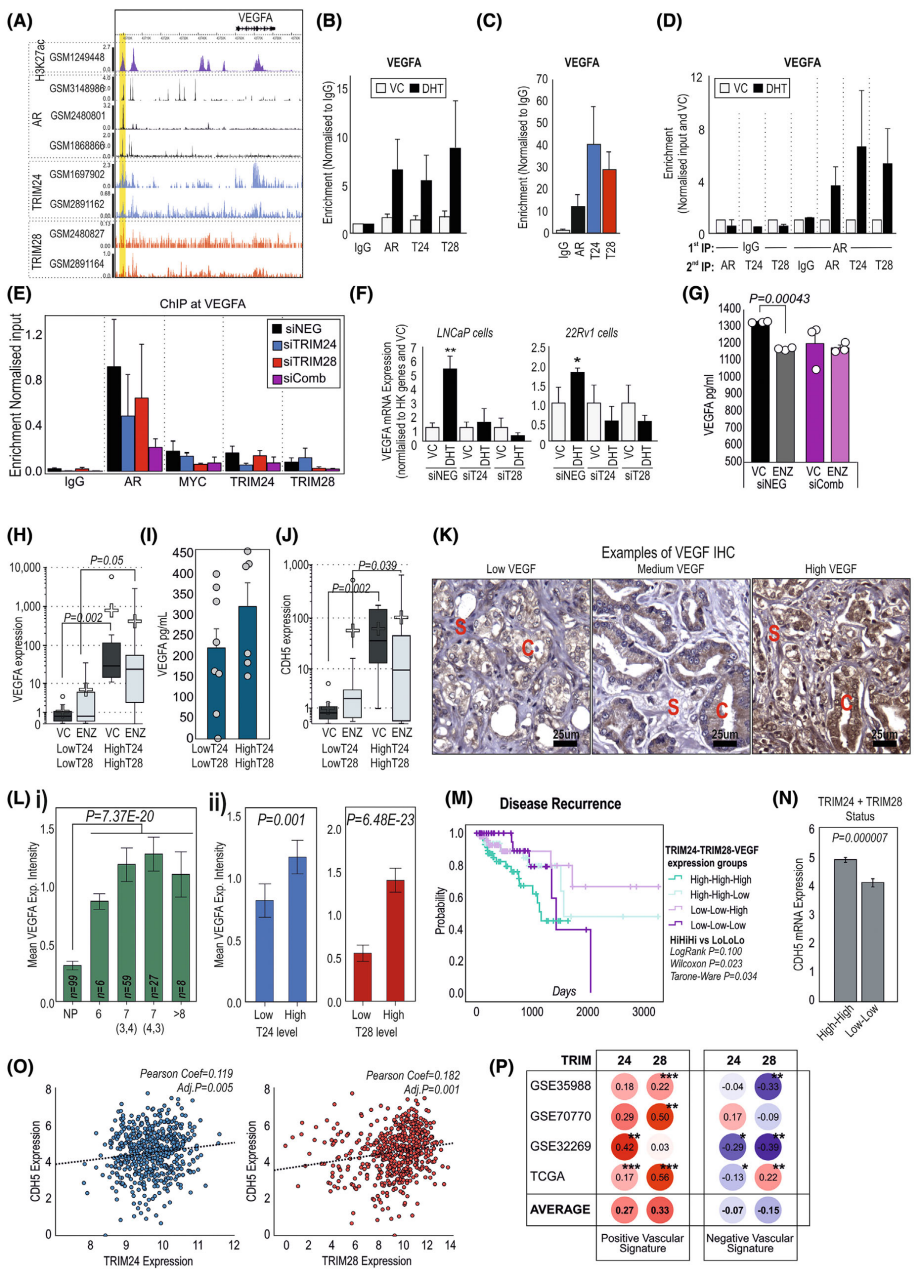
TRIM24 and TRIM28 expression associates with VEGFA in patient tissue. (A) Example of H3K27ac (GSM1249448 [113]), AR (GSM3148986, GSM2480801 [111], GSM1868866 (C4‐2B [34])), TRIM24 (GSM1697902 [22], GSM2891162 [25]), TRIM28 (GSM2480827 [111] and GSM2891164, [25]) binding in chromatin immunoprecipitation (ChIP)‐seq experiments completed in LNCaP cells. Yellow area indicates region primers for ChIP‐qPCR were designed for (B) ChIP‐qPCR of candidate genes in LNCaP cells treated with 10 nm Dihydrotestosterone (DHT) or equivalent vehicle control (VC). Immunoprecipitation (IP) was performed with AR, IgG, TRIM24 or TRIM28 and PCR for target regions in genes as indicated. Data are representative of five repeats normalised to input and IgG. Error bars indicate SEM. (C) RT‐qPCR from ChIP of AR, TRIM24 and TRIM28 completed in four prostate cancer samples (mean of *n* = 4 ± SEM shown) normalised to input and IgG. (D) Results from ChIP‐reChIP in LNCaP cells treated with 10 nm DHT or equivalent VC, where first IP was performed with either anti‐AR antibody or IgG, then a second IP for AR, TRIM24, TRIM28 or IgG. Presented as mean of *n* = 3 ± SD. (E) Results from CHIP in C4‐2B cells for AR, MYC, TRIM24 and TRIM28 binding to VEGFA. C4‐2B cells had been transfected with siRNA against TRIM24 and or TRIM28. Data is normalised to Input control and represent three biological repeats ± SD. (F) Expression of VEGFA in LNCaP and 22Rv1 cancer cells transfected with siRNA against TRIM24 and or TRIM28 and treated with 10 nm DHT or equivalent vehicle control (VC). RT‐qPCR data represent mean of *N* = 3, normalised to VC, ±SD. Significance determined via *t*‐test comparing DHT to VC treatment, * = *P* < 0.05, ** = *P* < 0.01. (G) Data from ELISA detecting VEGFA concentrations in conditioned media from C4‐2B cells transfected with siRNA against TRIM24 and TRIM28 compared to negative control. Cells were also treated with 10 μm Enzalutamide (ENZ) or vehicle control (VC). Data represent mean of three biological repeats ± SD. Significance determined via Student *t*‐test. (H) Expression of VEGF in prostate cancer PDE samples were divided based on TRIM expression, into those with high TRIM24 and TRIM28 expression (HighT24‐HighT28, *N* = 8) and those with low TRIM24 and TRIM28 expression (LowT24‐LowT28, *N* = 8). The expression of genes in samples treated with 10 μm enzalutamide (ENZ) or vehicle control (VC) for 72 h. Results were normalised to VC of Low‐Low samples. Depicted as boxplot of median, whiskers represent 1st and 4th quartile, with mean indicated as a cross. Significance was determined ANOVA. (I) VEGFA ELISA detecting secreted VEGFA in conditioned media from explant samples treated with 10 μm enzalutamide (ENZ). Samples divided in HighT24‐HighT28 (*n* = 8) and LowT24‐LowT28 (*N* = 7) expressing samples. Data presented as mean ± SEM. Significance determined via Student *t*‐test compared ENZ to VC. (J) CDH5 (endothelial cell marker) expression in explant tissue using same samples outlined in (H). Samples divided in HighT24‐HighT28 (*n* = 8) and LowT24‐LowT28 (*N* = 8) expressing samples. Data presented as box plot of median, whiskers represent 1st and 4th quartile, with mean presented as a cross. Significance determined via Mann–Whitney *U*‐test. (K) Example VEGFA IHC staining of PCa tissue in a 99‐patient tissue microarray (TMA). Areas of cancer cells (C) and stroma (S) are designated. Scale bar = 25 μm. (L) (i) Expression analysis of VEGFA protein across cancer tissue of different Gleason graded tissue and patient matched normal tissue. (ii) VEGFA expression in patients dichotomised based on TRIM24 (low *n* = 42, high *n* = 56) or TRIM28 (low *n* = 42, high *n* = 55) protein levels. Data presented as mean of each group, with error bars indicating SEM. Significance determined via Independent *T*‐Test. (M) Kaplan–Meier analysis of disease reoccurrence in TCGA dataset of 498 patients grouped according to TRIM24, TRIM28 and VEGF expression. (N) Expression of endothelial cell marker in TCGA dataset in patients divided into those that expressing high *TRIM24* and *TRIM28* (High‐High) and low *TRIM24* and *TRIM28* (Low‐Low). Error bars indicate SEM. Significance determined by Student *t*‐test. (O) Correlation of *CDH5* and *TRIM24* or *TRIM28* expression in TCGA dataset, as determined by Pearson correlation and adjusted *P*‐value. (P) Correlation of *TRIM24* and *TRIM28* with gene signatures positively associated with vascularisation of tissue, and a gene signature negatively associated with vascularisation of tissue. These signatures were investigated in four patient cohorts (each row). Within each circle is the Pearson coefficient, and *, **, *** depict *P*‐values < 0.05, < 0.01, < 0.001, respectively. Colour of circle depict direction of correlation, red = positive, blue = negative correlation.

Neo‐angiogenesis is important to cancer progression generally and has been proposed to play a role in CRPC progression [65, 66, 67]. It is a process mediated by the androgen‐regulated VEGF protein, and here we show VEGF to be a target gene of both TRIM24 and TRIM28, as well as AR. Publicly available data suggest chromatin interaction of AR, TRIM24, and TRIM28 to regions of H3K27ac around the *VEGFA* gene (Fig. 5A), which is confirmed with candidate ChIP experiments indicating increased enrichment of AR, TRIM24 and TRIM28 binding with DHT (Fig. 5B) and confirm binding in patient tissue (Fig. 5C). The relationship between AR and TRIMs was further highlighted with ChIP‐reCHIP experiments with TRIM24 and TRIM28 binding to regions that pulldown with the initial AR‐ChIP (Fig. 5D). Silencing both TRIM24 and TRIM28 reduced the binding of AR and MYC (Fig. 5E). Silencing TRIM24 or TRIM28 reduced the ability of DHT to upregulate VEGFA mRNA and protein (Fig. 5F). In ELISAs, conditioned media from C4‐2B cells with both TRIM24 and TRIM28 silenced exhibited less secreted VEGFA, that further was no longer regulated by enzalutamide (Fig. 5G). In PDE models from 19 patients, prostate cancer tissue with high *TRIM24* and *TRIM28* expression had significantly higher expression of *VEGFA* mRNA (Fig. 5H), as well as proliferation markers *Ki67* and *PCNA* (Fig. S5A), also *CDH5*, a marker of endothelial cells (Fig. 5H). These associations were seen in the presence or absence of enzalutamide. In conditioned media from the same explant samples, secreted VEGFA levels were higher in samples that expressed high levels of TRIM24 and TRIM28 (Fig. 5I). RNA for *CD45*, a marker of endothelial cells/vasculature, was also increased in samples with high levels of both *TRIM24* and *TRIM28* (Fig. 5J).

We also examined the expression of VEGFA in our 99‐patient PCa tissue cohort (Fig. 5K). There were varying intensities of VEGFA immunostaining and, in general, an increase in VEGFA intensity in PCa compared to matched adjacent normal tissue (Fig. S5Li), and strong correlation between VEGFA levels in PCa samples and the fold change in VEGFA staining between cancer and benign tissue (Fig. S5Bi). When we compared back to the staining scores for the TRIM proteins, we found a significant positive correlation between VEGFA and either TRIM protein (Fig. S5Bii); further, those patients with either high TRIM24 or high TRIM28 had higher VEGFA immunostaining intensity (Fig. S5Lii). In support of this, in published PCa datasets, we found *VEGFA* expression to be increased in CRPC compared to primary PCa (*P* = 4.04E‐11, Fig. S5C) and positive correlation of *TRIM24* and *TRIM28* RNA expression with *VEGFA* (Fig. S5D). Similar correlations were also found for *MYC*, a target of TRIM24/TRIM28/AR as well as being a known regulator of *VEGF* [68, 69, 70] (Fig. S5D).

Furthermore, in the TCGA PCa dataset, if we group patients based on the expression of *TRIM24*, *TRIM28* and *VEGFA*, those patients that have high expression of all three have the shortest time till disease relapse (Fig. 5M). Furthermore, in this TCGA cohort, endothelial marker *CDH5* expression was significantly associated with *TRIM24* and *TRIM28* expression (Fig. 5N,O). We also assessed two vascularisation gene signatures [40], a gene signature that associates positively with vascularisation (VI^+^) and a gene signature that associates negatively with vascularisation (VI^−^), in four datasets, finding significant positive correlations between *TRIM24* and the VI^+^ signature in two out of four datasets (remaining two have the same trend but not significant) and inverse correlations with the VI^−^ signature in two of four datasets (Fig. 5P and Fig. S6). *TRIM28* expression correlated significantly with VI^+^ in three cohorts, the remaining cohort has the same trend, and we saw a significant inverse correlation with the VI^−^ signature in two of the four datasets (Fig. 5P and Fig. S6). Given the role of VEGFA, these data support a role for TRIM24 and TRIM28 in angiogenesis.

### 3.6 Functional relevance of TRIM24 and TRIM28 in VEGF mediation of vasculature formation and sensitivity to antiandrogens

**Fig. 6:**
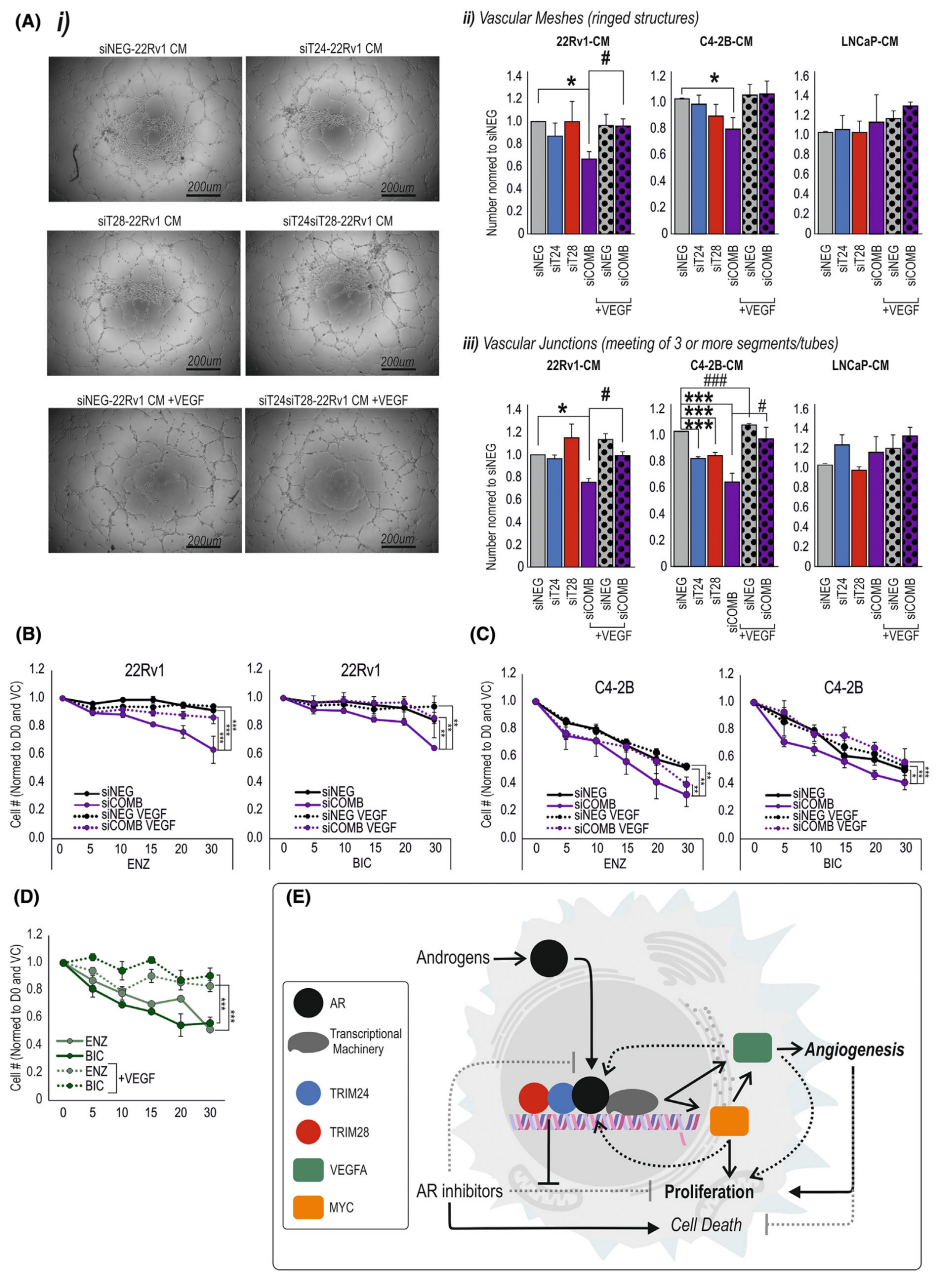
TRIM24 and TRIM28 expression regulates endothelial cell proliferation and angiogenesis. (A) Tube formation assay, measuring the ability of HUVEC cells to form angiogenic structures after 6 h in the presence of conditioned media (CM) from 22Rv1 cells transfected with siRNA against TRIM24 (siT24), TRIM28 (siT28) or a negative control siRNA (siNEG). Rescue effect was measured in the presence of recombinant VEGF. Controls of an inhibitor, no‐ECM and VEGF were also measured. (i) Example images of angiogenic structures. Controls are shown in Fig. S5F. Scale bar = 200 μm. (ii) Analysis of the number of angiogenic mesh structures formed, and (iii) the number of junctions between vascular tubes formed. Data are presented as the average of three experiments normalised to siNEG. Error bars indicate SD. * = *P* < 0.05, *** = *P* < 0.001 compared to siNEG, # = *P* < 0.05, ### = *P* < 0.001 compared to addition of VEGF. Statistics determined via Student *t*‐test. Proliferation of (B) 22Rv1 and (C) C4‐2B cells transfected with siRNA against TRIM24 and TRIM28 (siComb) or negative control (siNEG), treated with increasing doses of enzalutamide or bicalutamide in the presence or absence of VEGF. Depicted are mean cell numbers after 3 days of exposure to treatment, *n* = 3. Error bars indicate SD. Statistics are used to compare antiandrogen to VC and VEGF treatments. Significance determined via ANOVA, * = *P* < 0.05, ** = *P* < 0.01, *** = *P* < 0.001. (D) Proliferation of LNCaP cells exposed increasing doses of enzalutamide (ENZ) or bicalutamide (BIC) in the presence or absence of VEGF. Presented as mean of *n* = 4, with SEM. Significance determined via ANOVA, *** = *P* < 0.001. (E) Diagram of proposed mechanism for TRIM24 and TRIM28 in AR signalling and CRPC. As coregulators TRIM24 and TRIM28 combined to interact with and aid AR regulation of proliferation through upregulation of MYC, reducing enzalutamide‐induced cleaved caspase and upregulating VEGFA and angiogenesis.

To examine the potential functional relationship between TRIM24/TRIM28 and VEGFA and angiogenesis, we collected conditioned media (CM) from PCa cells in which either TRIM24 or TRIM28, or both, had been silenced, as well as from cancer cells treated with a scr‐siRNA (siNEG) as a control. We assessed whether silencing TRIMs affected the ability of cancer cell‐conditioned medium to influence HUVEC tube formation. Silencing both TRIM24 and TRIM28 in the 22Rv1 cells used to produce the CM reduced the number of ring structures formed by HUVEC cells and the number of branching points (Fig. 6Ai,ii,iii), both measures of angiogenesis. This effect could be rescued by the addition of exogenous VEGF to the conditioned media (Fig. 6Aii,iii). In C4‐2B cells, silencing both TRIM24 and TRIM28 produced CM that reduced the number of vascular meshes (Fig. 6Aii). Silencing TRIM24 or TRIM28, separately or together, reduced the number of vascular junctions, which could be rescued with exogenous VEGF (Fig. 6Aiii). Silencing TRIM24 or TRIM28 in LNCaP cells, however, did not significantly alter the formation of vascular meshes or junctions (Fig. 6Aii,iii). Additionally, the expression of the cell survival marker, *Ki67*, was also reduced in HUVEC cells treated with CM from TRIM24‐ or TRIM28‐silenced cancer cells as compared to control‐transfected cells (Fig. S5E).

We further investigated the relationship between TRIMs, VEGF and CRPC in the context of cancer cell proliferation. VEGF signalling has also been recently proposed as a mechanism by which PCa can become resistant to AR targeted therapy [71, 72]. In both 22Rv1 and C4‐2B cells, silencing both TRIM24 and TRIM28 together promoted sensitivity to enzalutamide and bicalutamide, as with previous experiments. Strikingly, the addition of exogenous VEGF re‐conferred resistance of 22Rv1 cells to both antiandrogens (Fig. 6B) and reduced the response of C4‐2B cells (Fig. 6C). VEGF alone did not significantly increase proliferation (Fig. S5G). In LNCaP cells, the addition of VEGF also was able to make cells significantly less responsive to both enzalutamide and bicalutamide, almost abolishing their response (Fig. 6D).

Together, these data suggest that TRIM24 and TRIM28 enhance AR upregulation of VEGFA secretion which may be able to stimulate vascular cell growth thus promoting angiogenesis. The effect of VEGFA appears to be two‐pronged, with a direct effect also on cancer cell proliferation, as illustrated by its ability to reverse the resensitisation (by TRIM knockdown) of cancer cells to antiandrogen therapies.

## 4 Discussion

From the data presented in this manuscript, we propose a model whereby increased expression of TRIM24 and TRIM28 in CRPC enables continued AR signalling (Fig. 6E). Specifically, this TRIM24/28‐influenced AR signalling upregulates oncogenic/proliferative genes such as MYC and VEGFA, culminating in enhanced PCa cell proliferation and reduced responsiveness to antiandrogens. We show that reducing both TRIM24 and TRIM28 can make therapy‐resistant CRPC cell line models sensitive to antiandrogens, which aligns with reports suggesting that MYC is involved in androgen sensitivity/castration resistance [42, 68, 69, 73, 74]. The requirement for both TRIM24 and TRIM28 to be silenced for this to occur suggests some functional redundancy between the two TRIMs. Importantly, we show that this resensitisation effect can be reversed by the addition of VEGF. TRIM‐mediated AR signalling and MYC are both able to regulate VEGF and blood vessel formation, as well as TRIM expression associating with vascularisation scores in patient tissues.

Given the clinical impact of therapy resistance in advanced PCa, there has been a growing movement to target the preserved AR signalling in CRPC through coregulators and interacting proteins, once AR‐directed therapies have failed. In terms of targeting coregulators, bromodomain‐containing coregulators have been of particular interest due to their druggability [75, 76]. There have also been developments in targeting TRIM proteins more specifically [20, 77, 78, 79, 80]. In our analysis of patient data, cell lines and animal models we find a consistent increase in TRIM24 and TRIM28 expression in PCa and progression, which is consistent with previous reports [22, 25, 81]. Previous studies have suggested interaction between TRIM24, TRIM28 and AR [13, 25], but here we show for the first time that this interaction exists between endogenous proteins in prostate cancer cells and occurs at chromatin to regulate DNA interactions and gene transcription. In androgen‐insensitive 22Rv1 and C4‐2B models of CRPC, silencing either TRIM24 or TRIM28 did not make cells sensitive to enzalutamide, but simultaneously silencing both TRIM24 and TRIM28 made these antiandrogen‐resistant cells sensitive. We have shown that there is overlap between the targets of TRIM24 and TRIM28, suggesting that one may compensate for the other in terms of influencing AR signalling, a possible explanation for this redundancy in terms of effect on antiandrogen response. Our data support the growing paradigm of targeting the interaction between key coregulators and AR as a means of further impeding AR activity when treating patients with advanced disease [82, 83, 84, 85, 86]. It also suggests that redundancy is perhaps an overlooked mechanism in coregulatory studies: coregulators may be interchangeable, to a degree, in mediating NR‐mediated transcription, and targeting a family/subgroup of coregulators may be needed to see a full effect.

Mechanistically, the effect of targeting TRIM24 and TRIM28 may involve effects on proliferation. Here we found an association between the TRIM status of cells and tissue with proliferation markers and drivers of proliferation such as MYC and its target cyclin D1, which both independently and as an AR‐regulated factor can control aggressive proliferation and survival [87, 88, 89, 90]. Interestingly, analysis of MYC ChIP‐seq in LNCaP cells shows there is MYC binding around the promoters of both TRIM24 and TRIM28 genes [91], which may point to regulatory/feedback loops. We do also see an association between TRIM24 and TRIM28 expression in explant models and gene silencing, with heightened cleaved caspase response to enzalutamide, which may involve TRIMs association with P53, as P53 mediated apoptosis is reported to be independently influenced by either TRIM24 [92, 93, 94] or TRIM28 [95, 96, 97, 98], with our complementary results suggesting that having high expression of both may protect PCa cells against antiandrogen induced apoptosis.

TRIM24 and TRIM28 have reported roles in mediating angiogenesis [99, 100, 101], and in other tumour types, inhibition of bromodomain proteins has been associated with inhibiting angiogenesis [102]. Here we find regulation of the AR target and angiogenic factor VEGFA by TRIM24 and TRIM28, which produced subsequent effects on endothelial cell proliferation and tube formation. Androgens are known to regulate angiogenesis [65, 103], with an androgen response element present within the promoter of VEGFA [104]. MYC is also known to influence tumour vascularisation [105] and the link between TRIMs and MYC, TRIMs and VEGF, respectively, may reflect a functional association with TRIMs (±AR) being part of MYC regulation of VEGFA. Although targeting VEGF/VEGF‐receptors has had conflicting results in clinical trials [106], targeting angiogenesis has been reported recently to be a viable means of blocking growth in human xenograft models of CRPC [107]. Furthermore, combining ADT with the VEGF inhibitor, Bevacizumab, has been reported to enhance PCa patient survival [108], suggesting that targeting angiogenesis may have an important role in fighting CRPC. Both TRIM24 and TRIM28 have been reported to have nontranscriptional roles in regulation pathways involved in angiogenesis including influencing DNA‐damage responses [94], influencing transduction signalling pathways [109], as well as interactions with SPOP [25], which itself has been reported to regulate VEGF signalling proteins [110], thus there is opportunity for the effects of TRIM24 and TRIM28 on vascularisation to be more extensive. Given our findings in this study, further investigation is warranted into the field of coregulator‐mediated angiogenesis as a means of inhibiting CRPC and cancer progression.

## 5 Conclusion

AR coregulators, TRIM24 and TRIM28, combine to influence angiogenesis and responsiveness to antiandrogen treatment.

## Conflict of interest

The authors declare no conflict of interest.

## Author contributions

DAL: study design, experimentation, analysis, interpretation, manuscript preparation and editing; NC and KS: experimentation; GSA and AV‐C: experimentation, manuscript editing; TTS, MW and HUA: patient tissue sampling/collection; CLB: conceptualisation, study design, interpretation, manuscript editing. All authors reviewed and approved the manuscript.

## Supporting information


**Fig. S1.** Further analysis of TRIM proteins in clinical data.
**Fig. S2.** Silencing TRIM24 and TRIM28, further effects on expression, proliferation, and interaction with chromatin.
**Fig. S3.** Silencing TRIM24 and TRIM28 effects on DHT responses and regulation of MYC.
**Fig. S4.** Silencing TRIM24 and TRIM28 effects on response to anti‐androgens and bromodomain inhibitors.
**Fig. S5.** Further associations between TRIM proteins and VEGF and angiogenesis.
**Fig. S6.** Association between TRIM24 and TRIM28 with vascularisation signatures in clinical datasets.

**Table S1.** Primer sequences.

**Table S2.** Coregulator expression in CRPC cohorts.

## Data Availability

Data that support the findings of this study are openly available in NCBI's Gene Expression Omnibus and are accessible via https://www.ncbi.nlm.nih.gov/geo, with the following GEO series accession numbers: ChIP: GSE27823, GSE51621, GSE94682, GSE114732, GSE72714, GSE69331, GSE108144, GSE94682, GSE108144. Microarray data: GSE35988, GSE70770, GSE32269. RNA seq data from TCGA cohort are available at https://www.cbioportal.org/study/summary?id=prad_tcga.
