# Supplementary material for: Simultaneous inhibition of TRIM24 and TRIM28 sensitises prostate cancer cells to antiandrogen therapy, decreasing VEGF signalling and angiogenesis
Source: Mol Oncol. 2025 May 24;19(10):2797–821. doi: 10.1002/1878-0261.70065 (PMC12515719; doi:10.1002/1878-0261.70065)
Supplement: Supplementary file 1 — Fig. S1. Further analysis of TRIM proteins in clinical data. Fig. S2. Silencing TRIM24 and TRIM28, further effects on expression, proliferation, and interaction with chromatin. Fig. S3. Silencing TRIM24 and TRIM28 effects on DHT responses and regulation of MYC. Fig. S4. Silencing TRIM24 and TRIM28 effects on response to anti‐androgens and bromodomain inhibitors. Fig. S5. Further associations between TRIM proteins and VEGF and angiogenesis. Fig. S6. Association between TRIM24 and TRIM28 with vascularisation signatures in clinical datasets. [file MOL2-19-2797-s002.zip › mol270065-sup-0001-Figures.pdf]

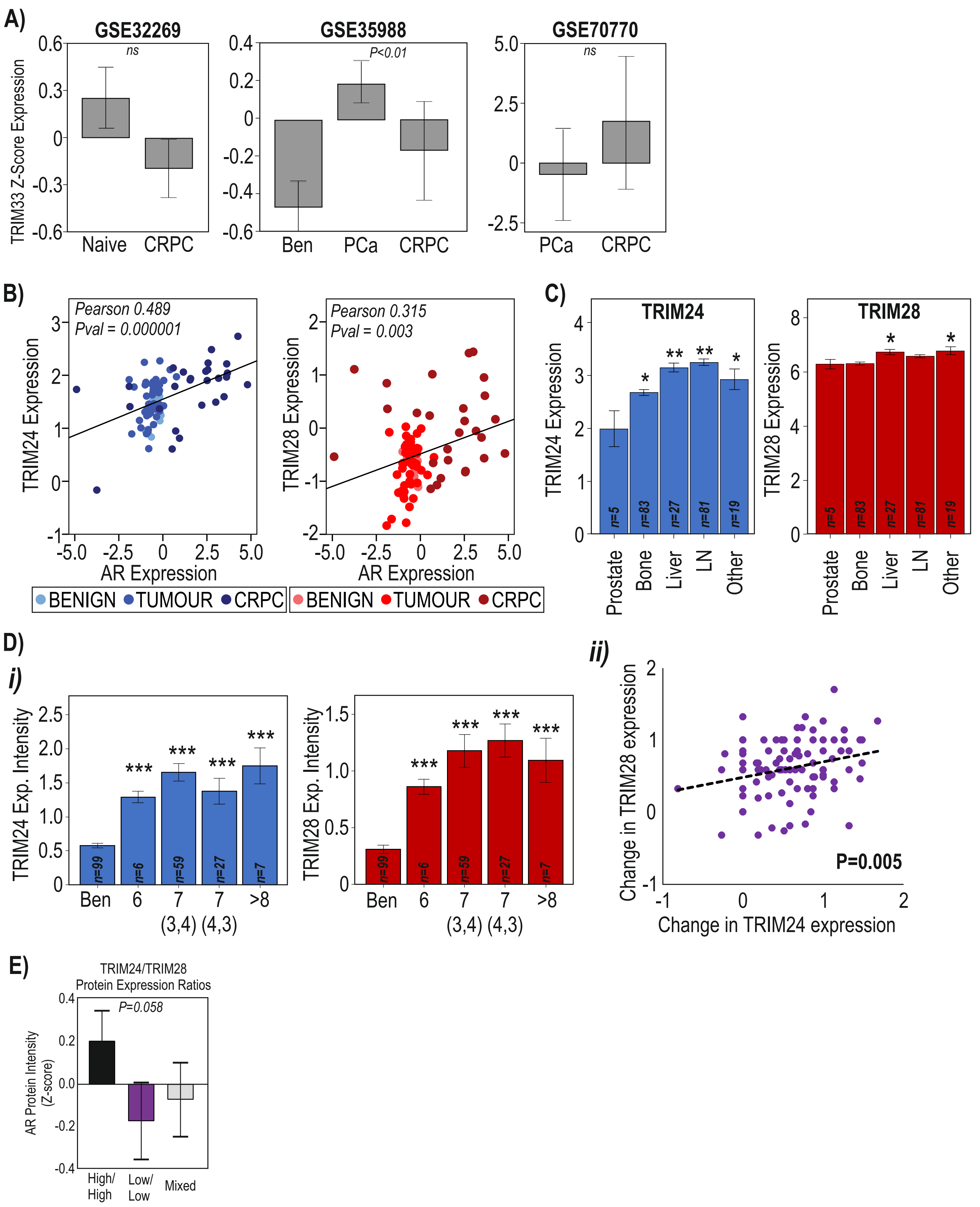

SUPPLEMENTARY FIGURE 1

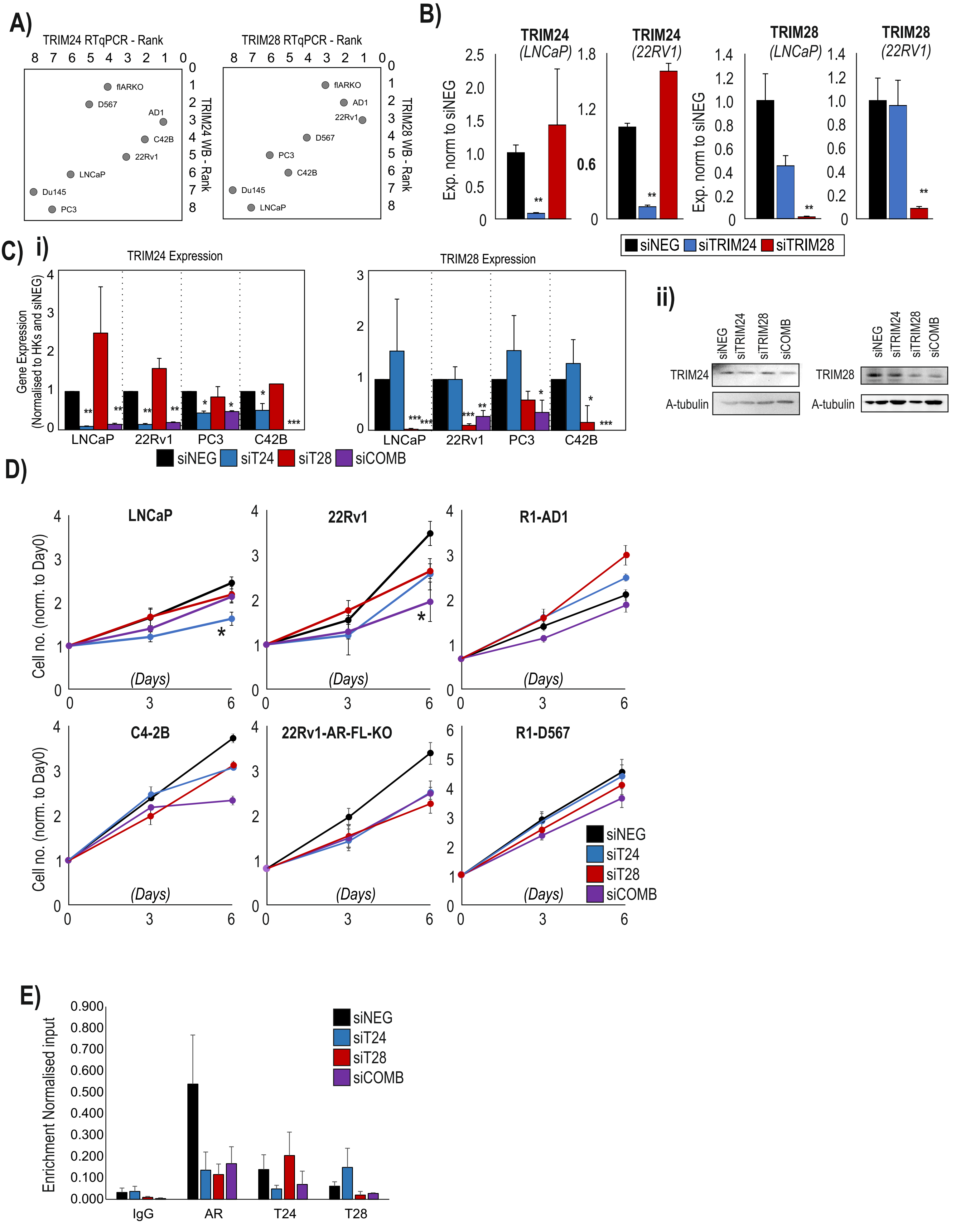

SUPPLEMENTARY FIGURE 2

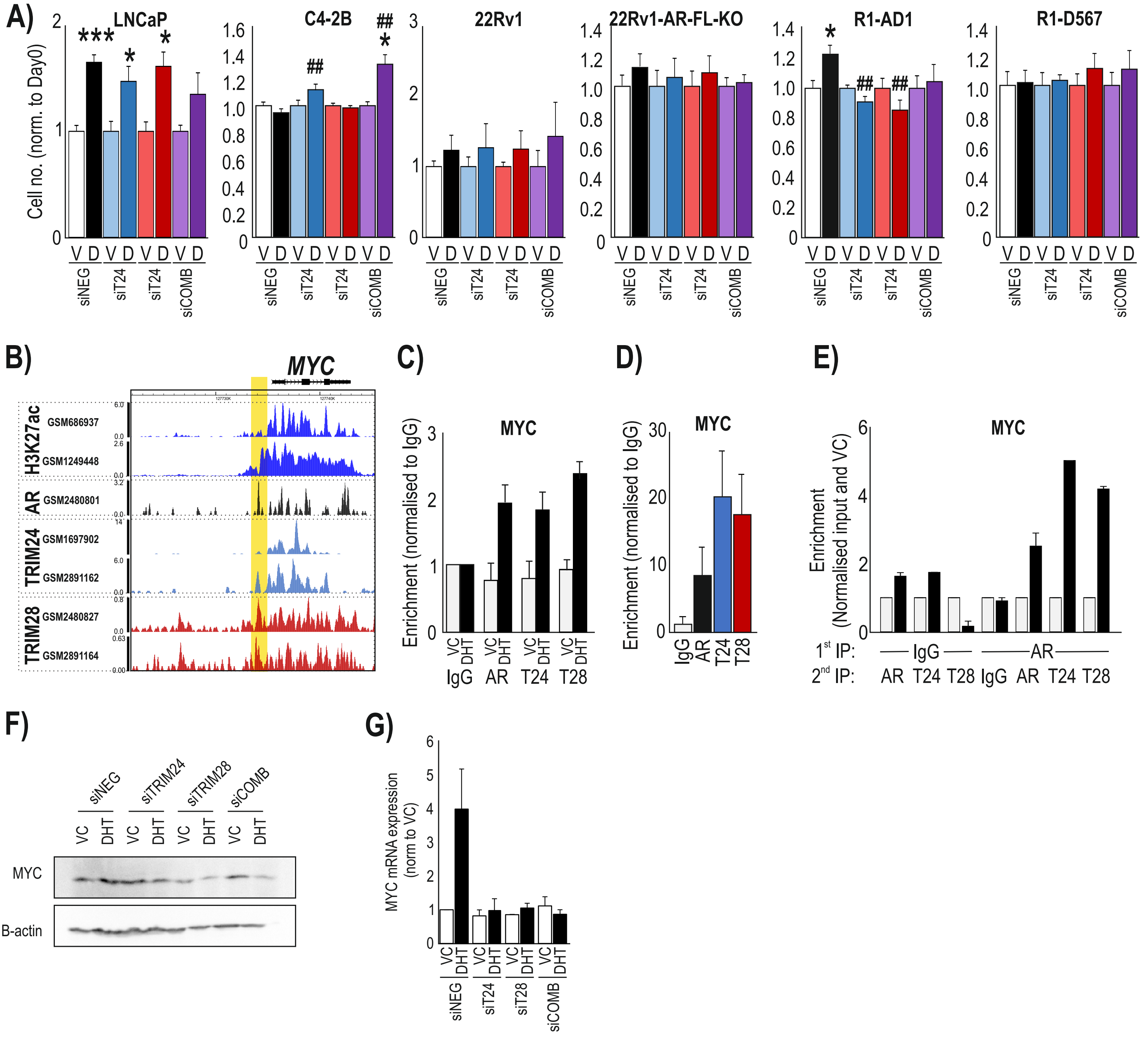

SUPPLEMENTARY FIGURE 3

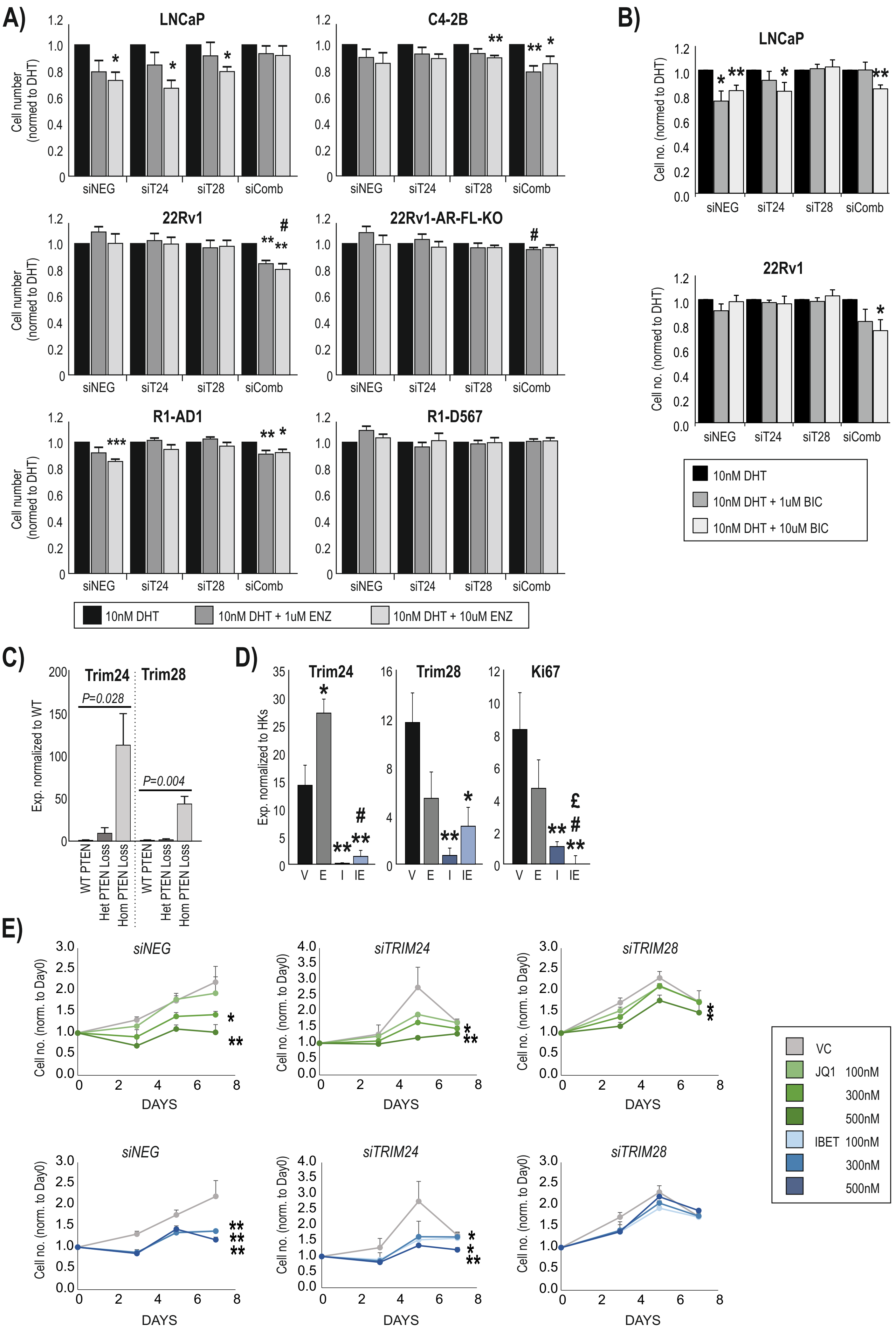

SUPPLEMENTARY FIGURE 4

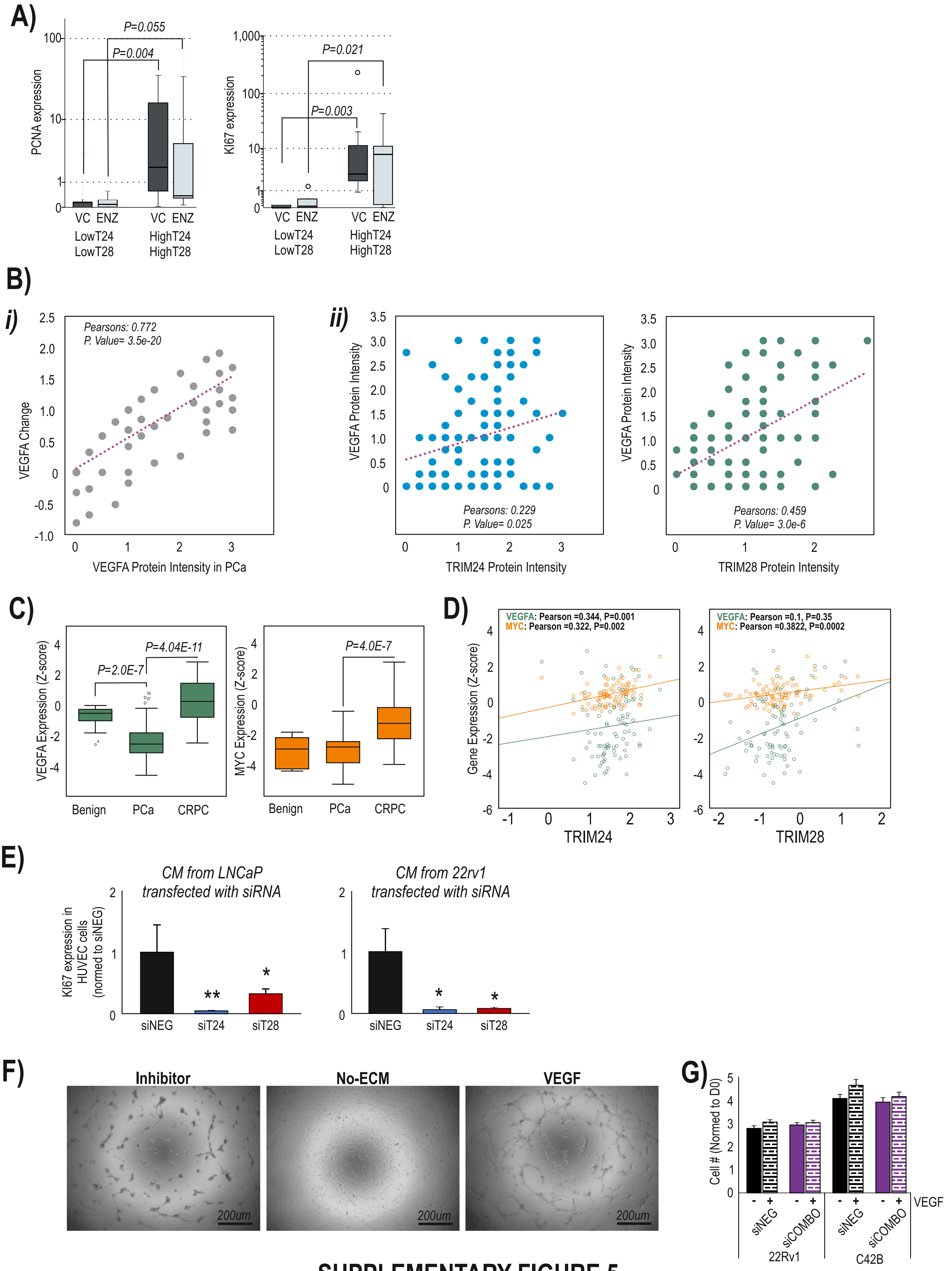

SUPPLEMENTARY FIGURE 5

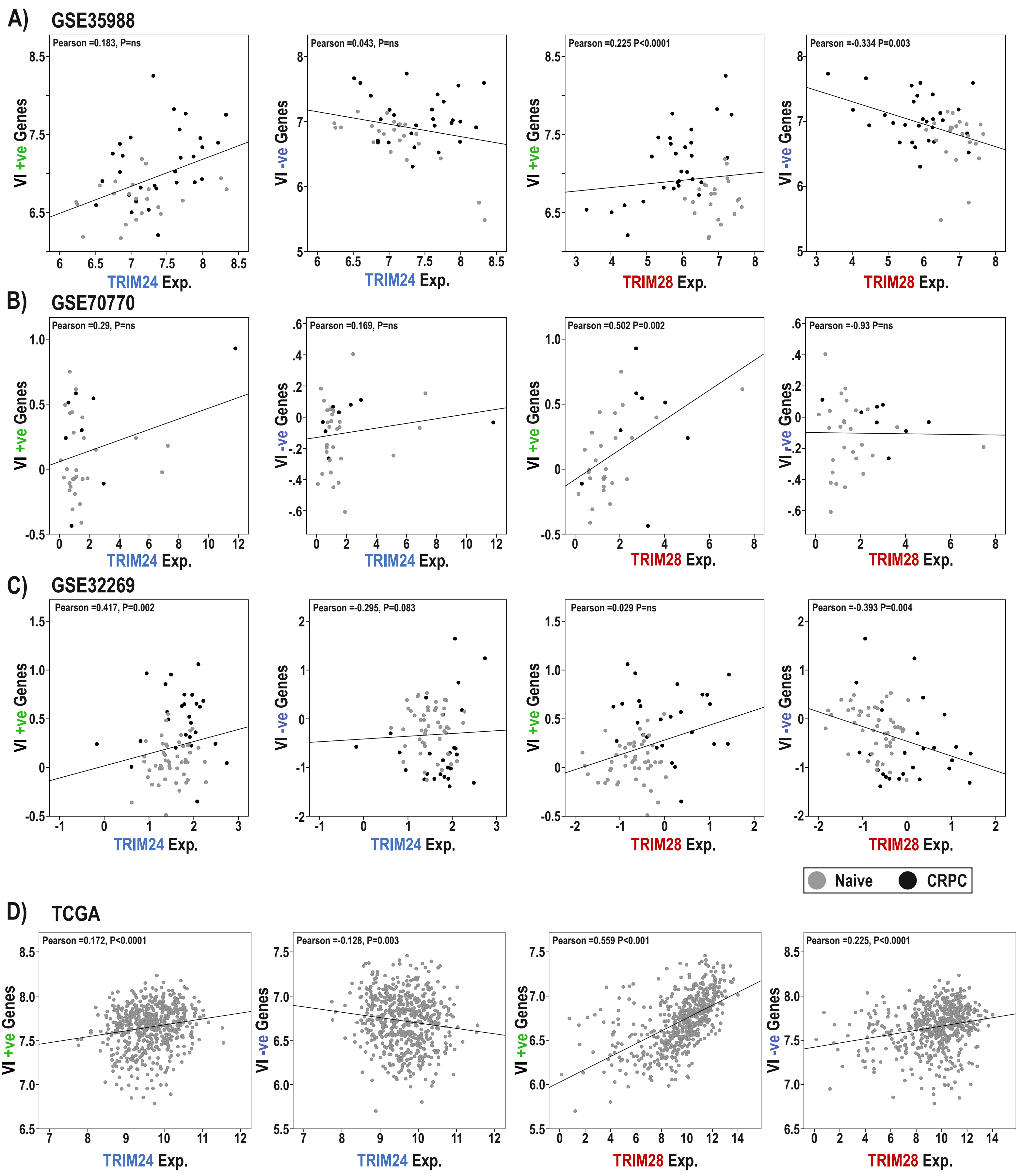

SUPPLEMENTARY FIGURE 6
