# Supplementary material for: Simultaneous inhibition of TRIM24 and TRIM28 sensitises prostate cancer cells to antiandrogen therapy, decreasing VEGF signalling and angiogenesis
Source: Mol Oncol. 2025 May 24;19(10):2797–821. doi: 10.1002/1878-0261.70065 (PMC12515719; doi:10.1002/1878-0261.70065)
Supplement: Supplementary file 1 — Fig. S1. Further analysis of TRIM proteins in clinical data. Fig. S2. Silencing TRIM24 and TRIM28, further effects on expression, proliferation, and interaction with chromatin. Fig. S3. Silencing TRIM24 and TRIM28 effects on DHT responses and regulation of MYC. Fig. S4. Silencing TRIM24 and TRIM28 effects on response to anti‐androgens and bromodomain inhibitors. Fig. S5. Further associations between TRIM proteins and VEGF and angiogenesis. Fig. S6. Association between TRIM24 and TRIM28 with vascularisation signatures in clinical datasets. [file MOL2-19-2797-s002.zip › Supp Figure Legends 2025.docx]

**Supplementary Figure 1. Further analysis of TRIM proteins in clinical data.**

**A)** Z-Score expression of TRIM33 in three datasets comparing CRPC to non-CRPC (PCa) and benign (Ben) samples **B)** Correlation between AR and TRIM24 or TRIM28 in benign, primary tumour (tumour), and metastatic (CRPC) prostate cancer samples (GSE35988). Correlation determined by Pearson Coefficient. **C)** Expression of TRIM24 and TRIM28 RNA in metastatic PCa lesions at different sites (GSE6919 LN = lymph node). Significance between metastatic sites and primary prostate cancer tested with Student T-Test, *<0.05, **<0.01. **D)** Further comparisons for immunohistochemical staining of TRIM24 or TRIM28 in a TMA of 99 patients with cores from both cancer and matched benign (Ben) tissue from each patient: *i)* Expression intensity scores for TRIM24 and TRIM28 were compared between Adjacent normal and Gleason grades (significance shown between individual grades compared to normal tissue, ***P<0.001)), *ii)* Correlation between TRIM24 and TRIM28 staining changes in PCa samples compared to adjacent normal. **E)** AR protein expression scores in TMA, grouped according to High expression of TRIM24 and TRIM28, compared to low expression of both, or mixed low and high of either.

**Supplementary Figure 2. Silencing TRIM24 and TRIM28, further effects on expression, proliferation, and interaction with chromatin.**

**A)** Dot plots of TRIM24 and TRIM28, comparing rank expression at mRNA and protein levels in prostate cancer cell lines. **B*)*** RNA expression of TRIM24 and TRIM28 in LNCaP and 22RV1 cells transfected with siRNA (72-hours post transfection, n=3). **C) i)** Bar graphs representing change in TRIM24 or TRIM28 expression in multiple cells lines after transfection siRNA against TRIM24, TRIM28, or cells transfected with siRNA against both. Student T-Test was used to compare expression between siRNA transfected cells and the siRNA negative control (siNEG). * = P<0.05, ** =P<0.01, ***=P<0.001. **ii)** Example immunoblot of C4-2B cell transfected with siRNA against TRIM24, TRIM28, and a combination of both, compared to siNEG control**. D)** MTT assays measuring proliferation of LNCaP, C4-2B, 22RV1, 22RV1-ARflKO, R1-AD1, R1-D567 cells after silencing TRIM24 (siT24) or TRIM28 (siTRIM28) alone or in combination. Cells transfected with a negative control are denoted as siNEG. **E)** Results from ChIP in C42B cells for AR, TRIM24, and TRIM28 binding to FKBP5. C42B cells had been transfected with siRNA against TRIM24 and or TRIM28. Data is normalised to Input control, and represent three biological repeats***.***

**Supplementary Figure 3. Silencing TRIM24 and TRIM28 effects on DHT responses and regulation of MYC**

***A)*** Day 6 MTT assays measured proliferation in the presence of 10nM DHT or equivalent vehicle control (VC). Data normalised to VC for each siRNA condition. ***B)*** Example of H3K27ac (GSM686937, GSM1249448), AR (GSM2480801), TRIM24 (GSM1697902, GSM2891162), TRIM28 (GSM2480827, GSM2891164,) binding in ChIP-seq experiments completed in LNCaP cells. Yellow area indicates coding area of gene of interest. ***C)*** ChIP-PCR of MYC regulatory regions in LNCaP cells treated with 10nM DHT or equivalent VC. IP was performed with AR, IgG, TRIM24 or TRIM28 and PCR for target regions in genes as indicated. Data are representative of 5 repeats normalised to input and IgG. ***D)*** RT-qPCR from ChIP of AR, TRIM24, TRIM28 completed in four prostate cancer samples (mean ± SEM shown) normalised to input and IgG. ***E)*** Results from ChIP-reChIP at the MYC regulatory region in LNCaP cells treated with 10nM DHT or equivalent VC, where first IP was performed with either anti-AR antibody or IgG, then a second IP for AR, TRIM24, TRIM28, or IgG. ***F)*** Immunoblot analysis of MYC protein expression in cells transfected with siRNA against TRIM24, TRIM28, or a negative control (siNEG), treated with 10nM DHT or equivalent VC. (same membrane as Fig3F) ***G)*** RT-qPCR analysis of MYC gene expression in cells transfected with siRNA against TRIM24, TRIM28, or a negative control (siNEG), treated with 10nM DHT or equivalent VC. Expression normalised to housekeeping genes then VC treatment. Represents mean of N=3.

**Supplementary Figure 4. Silencing TRIM24 and TRIM28 effects on response to anti-androgens and bromodomain inhibitors.**

***A)*** MTT assays measuring proliferative response of LNCaP, C4-2B, 22RV1, 22RV1-ARflKO, R1-AD1, R1-D567 cells to enzalutamide (ENZ) after silencing TRIM24 (siT24) or TRIM28 (siTRIM28) alone or in combination (siComb) after 6 days. Cells transfected with a negative control are denoted as siNEG. ***B)*** MTT assays measuring proliferative response of LNCaP and 22RV1 cells to bicalutamide (BIC) after silencing TRIM24 (siT24) or TRIM28 (siTRIM28) alone or in combination (siComb) after 6 days. Cells transfected with a negative control are denoted as siNEG. Significance was determined by Student T-test, *, **, *** = P<0.05, P<0.01, P<0.001compared to corresponding DHT. # = P<0.05 compared to siNEG. ***C)*** RT-qPCR analysis of gene expression in PTEN-homozygous loss mice, treated with VC (V), Enzalutamide (E),and I-BET (I) alone or in combination (IE). **D)** Expression of Trim24, Trim28, and Ki67 in the prostate with PTEN-homozygous loss. Significance compared to VC determined with Student T-Test, *=P<0.05, **=P<0.01, significance between IE to E denoted as #= P<0.05, and significance between IE and I denoted as £ =P<0.05. ***E)*** Proliferation of LNCaP cells transfected with siRNA against TRIM24, TRIM28, or negative control, then treated with increasing doses of JQ1 and IBET. ***E)***. Significance between drug treatments to DHT were determined via Student T-Test, *P<0.05, **P<0.01, ***P<0.001, significance between siRNA samples and siNEG denoted as # P<0.05. Significance between co-treatment IE to E denoted as # P<0.05 or to VC as £ P<0.05.

**Supplementary Figure 5. Further associations between TRIM proteins and VEGF and angiogenesis.**

***A)*** Analysis of target genes in PDE of PCa patient tissue treated with 10uM Enzalutamide (ENZ) or equivalent vehicle control (VC) for three days. Sample were grouped based on high or low expression of bother TRIM24 and TRIM28 genes. Expression of mRNA of PCNA and KI67 genes was assessed, represented as boxplots, statistics calculated with Kruksal-Wallis.  ***B)*** Further analysis of immunohistochemical staining of a cohort of 99 PCa patients *i****)*** Correlation between the changes in VEGFA expression (between PCa and patient matched benign samples) with VEGFA protein expression score in PCa samples. ii) Correlation between the *VEGFA* mRNA expression and *TRIM24* and *TRIM28* mRNA expression in PCa samples. ***C)*** VEGFA and MYC genes expression in Benign, Primary PCa, and CRPC tissue (GSE35988). ***D)*** Correlation of VEGFA and MYC gene expression with TRIM gene expression in Cancer and CRPC samples from GSE35988. ***E)*** Expression of ki67 proliferation marker in HUVECs treated with conditioned media (CM) from LNCaP and 22RV1 cells transfected with siRNA against *TRIM24*, *TRIM28*, or a scramble control. Significance between cells treated with CM from siNEG and siRNA transfected cells determined via Student T-Test, * P<0.05, ** P<0.01. ***F)*** Example images of controls used in tube formation assays**. G*)*** Proliferation data from 22Rv1 and C42B cells transfected with siRNA against both TRIM24 and TRIM28 or negative control, in the presence or absence of exogenous VEGF (10 ng/mL). Data represents mean of 3 independent repeats.

**Supplementary Figure 6. Association between TRIM24 and TRIM28 with vascularisation signatures in clinical datasets.**

Correlation studies of TRIM24/28 genes with two gene signatures, one that positively associates with Vascularisation of tumours (VI+ve) and one that negatively associates with vascularisation of tumours (VI-ve). Analysis was carried out in four datasets three of which include CRPC samples, namely **A)** GSE35988 (n=226), **B)** GSE70770 (n=135), **C)** GSE32269 (n=119), and also **D) a** dataset of primary PCa (TCGA, n=499).
