# Supplementary material for: Simultaneous inhibition of TRIM24 and TRIM28 sensitises prostate cancer cells to antiandrogen therapy, decreasing VEGF signalling and angiogenesis
Source: Mol Oncol. 2025 May 24;19(10):2797–821. doi: 10.1002/1878-0261.70065 (PMC12515719; doi:10.1002/1878-0261.70065)
Supplement: Supplementary file 2 — Table S1. Primer sequences. [file MOL2-19-2797-s001.docx]

|  |  |  |  |  |  |
| --- | --- | --- | --- | --- | --- |
|  | **RT-QPCR** | |  | **Forward** | **Reverse** |
|  |  | FKBP5 |  | ATTATCCGGAGAACCAAACG | CAAACATCCTTCCACCACAG |
|  |  | BACTIN |  | GGCATCCTCACCCTGAAGTA | GGTCATCTTCTCGCGGTTG |
|  |  | GAPDH |  | ATGGGGAAGGTGAAGGTCG | GGGGTCATTGATGGCAACAATA |
|  |  | L19 |  | GCGGAAGGGTACAGCCAAT | AGCAGCCGGCGCAAA |
|  |  | KI67 |  | CGTCCCAGTGGAAGAGTTGT | CGACCCCGCTCCTTTTGATA |
|  |  | PCNA |  | GGCCGAAGATAACGCGGATAC | GGCATATACGTGCAAATTCACCA |
|  |  | KLK3/PSA |  | TTGTCTTCCTCACCCTGTCC | AGCTGTGGCTGACCTGAAAT |
|  |  | VEGFA |  | TGGAGCGTGTACGTTGGTG | GCGAGTCTGTGTTTTTGCAG |
|  |  | MYC |  | GTCAAGAGGCGAACACACAAC | TTGGACGGACAGGATGTATGC |
|  |  | CDH5/VECAD |  | CTTCACCCAGACCAAGTACACA | AATGGTGAAAGCGTCCTGGT |
|  |  | TRIM24 |  | CCCTCAAACAGAACGGTCCA | CTGGAGGCACTAGGTGAACG |
|  |  | TRIM28 |  | GTGAGACCTGTGTAGAGGCG | ACAGTACGTTCACCATCCCG |
|  |  |  |  |  |  |
|  | **ChIP-QPCR** | |  | **Forward** | **Reverse** |
|  |  | FKBP5 |  | GGGGGCAGTGGAGGAGAGGG | GCACAGCGTGTGGCCTGTCT |
|  |  | KLK3/PSA |  | GTGCATCCAGGGTGATCTAGTAATT | CACACCCAGAGCTGTGGAA |
|  |  | VEGFA |  | CCCATCCCCTCTTCATTTCT | GCAGCTCTTCTGGGACTTGT |
|  |  | MYC |  | ACACTAACATCCCACGCTCTG | GATCAAGAGTCCCAGGGAGA |
|  |  |  |  |  |  |

Supplementary table 1: Sequences of primers used.
