# Supplementary material for: Simultaneous inhibition of TRIM24 and TRIM28 sensitises prostate cancer cells to antiandrogen therapy, decreasing VEGF signalling and angiogenesis
Source: Mol Oncol. 2025 May 24;19(10):2797–821. doi: 10.1002/1878-0261.70065 (PMC12515719; doi:10.1002/1878-0261.70065)
Supplement: Supplementary file 3 — Table S2. Coregulator expression in CRPC cohorts. [file MOL2-19-2797-s003.docx]

|  | **GSE33269** | | **GSE33269** | | **GSE70770** | |
| --- | --- | --- | --- | --- | --- | --- |
|  | **LFC** | ***adjp*** | **LFC** | ***adjp*** | **LFC** | ***adjp*** |
| **AES** | -0.50702 | *0.060711* | 0.91657 | *0.018019* | -0.07227 | *0.672102* |
| **AIP** | -0.50403 | *0.022106* | -0.15837 | *0.687688* | 0.18712 | *0.191228* |
| **AKAP13** | -0.31926 | *0.156151* | -0.10594 | *0.789546* | 0.018145 | *0.978315* |
| **AKR1C1** | 2.100846 | *3.92E-07* |  |  | -0.29797 | *0.428809* |
| **AKR1C2** |  |  | 0.517567 | *0.183175* |  |  |
| **AKR1C3** | 2.036402 | *2.84E-05* | 0.559946 | *0.174795* | 0.043456 | *0.942341* |
| **AKT1** | -0.25325 | *0.350492* | 0.771452 | *0.047019* | 0.122063 | *0.463872* |
| **ANKRD11** | 0.853092 | *0.001284* |  |  | 0.491102 | *0.000128* |
| **ANP32A** | -0.45027 | *0.008041* |  |  | -0.70804 | *9.34E-08* |
| **APOBEC1** | -0.01 | *0.976406* | -0.33933 | *0.384004* | 3.317 | *1* |
| **APOBEC2** | 0.203535 | *0.354991* | 0.383724 | *0.336168* | -1.09467 | *0.037534* |
| **APOBEC3A** | 0.420044 | *0.210849* | 1.283092 | *0.000987* | -0.76496 | *0.099161* |
| **APOBEC3B** | 0.485476 | *0.049008* | 0.450946 | *0.260002* | -0.00273 | *1* |
| **APOBEC3C** | 0.120946 | *0.711895* | -0.36891 | *0.327323* | -0.98874 | *0.000122* |
| **APOBEC3D** |  |  |  |  | 0.008253 | *0.996619* |
| **APPBP2** | -0.50442 | *0.020875* | 0.244791 | *0.538439* | -1.29492 | *1.59E-06* |
| **APPL1** | -0.05673 | *0.837941* | 0.062103 | *0.874244* | -0.38576 | *0.067404* |
| **AR** | 2.414544 | *2.56E-09* | 0.858851 | *0.033279* | 2.097896 | *3.26E-07* |
| **ARID1A** | 1.491008 | *3.29E-07* | 0.905272 | *0.022099* | 0.71055 | *0.000227* |
| **ARID1B** |  |  | -0.28963 | *0.448288* | 0.878091 | *9.87E-09* |
| **AURKA** | 1.929157 | *5.48E-10* |  |  | 1.925437 | *6.92E-13* |
| **BAG1** | -0.43657 | *0.009984* |  |  | -0.74974 | *1.26E-07* |
| **BCAS2** | -0.23776 | *0.210422* |  |  | -1.39238 | *2.59E-10* |
| **BCAS3** | -0.25274 | *0.148889* | -0.47365 | *0.235068* | -0.37415 | *0.000169* |
| **BCL11A** | -0.29778 | *0.248021* | -0.2642 | *0.497315* | -1.53421 | *0.001169* |
| **BCL11B** | 0.008249 | *0.980322* | -0.25849 | *0.50803* | 0.412633 | *1* |
| **BCL3** | -0.50021 | *0.097312* | 0.165574 | *0.66798* | 0.567508 | *0.00409* |
| **BLOC1S1** |  |  |  |  | 0.081239 | *0.659293* |
| **BRCA1** | 0.919068 | *0.000123* | 0.892195 | *0.025492* | 1.181044 | *7.01E-11* |
| **BRCA2** | 0.593804 | *0.006319* | -0.23638 | *0.540627* |  | *1* |
| **BRD8** | 0.664788 | *1.84E-05* | -0.66339 | *0.09699* | 0.002049 | *1* |
| **CALCOCO1** | 0.636219 | *0.002221* | 0.12661 | *0.740564* | 0.189918 | *0.120171* |
| **CALR** | -0.24346 | *0.155245* |  |  | 0.409188 | *0.003951* |
| **CARM1** | 0.314677 | *0.10971* | 0.494989 | *0.205797* |  |  |
| **CASP8AP2** | 0.616493 | *0.013556* |  |  | -0.46426 | *0.010539* |
| **CAV1** | 0.204405 | *0.612328* | -0.40029 | *0.303893* | -3.03285 | *4.72E-14* |
| **CCNA1** | 0.363812 | *0.140421* |  |  | 2.755932 | *1* |
| **CCNA2** | 1.6867 | *1.26E-11* | 1.558324 | *0.000101* | 1.782561 | *2.07E-10* |
| **CCND1** | 0.705353 | *0.024618* | 0.422312 | *0.275739* | -0.07535 | *0.802077* |
| **CCND3** | 0.154939 | *0.356928* | -0.00667 | *0.986425* | 0.121388 | *0.511658* |
| **CCNE1** | 1.273402 | *5.96E-07* | 0.526581 | *0.182344* | 1.138403 | *6.28E-11* |
| **CDC25A** | 1.01115 | *0.000407* | -0.11158 | *0.776321* | 0.352905 | *0.145402* |
| **CDC25B** | 0.719121 | *0.000468* | 0.235711 | *0.547049* | 0.984902 | *5.38E-09* |
| **CDC7** | 1.230819 | *1.23E-09* | 0.525016 | *0.178354* | 1.420766 | *2.80E-08* |
| **CDK5** | -0.08387 | *0.84071* |  |  | 0.262741 | *0.061776* |
| **CDK6** | 0.05591 | *0.853701* |  |  | -1.62063 | *1.28E-05* |
| **CDK7** |  |  |  |  | -0.53595 | *0.000927* |
| **CDK9** |  |  | 0.443523 | *0.255554* | 0.649604 | *0.019309* |
| **CDKN1C** | -0.42463 | *0.09062* |  |  | -0.47477 | *0.018559* |
| **CDKN3** | 1.261087 | *1.01E-05* | 1.601168 | *5.35E-05* | 1.679054 | *1.44E-06* |
| **CDT1** |  |  |  |  | 1.951373 | *1.25E-13* |
| **CFL1** | 0.386599 | *0.075242* |  |  | 0.330803 | *0.090555* |
| **CITED1** | 0.194989 | *0.555464* | 1.093343 | *0.006494* | -1.03171 | *0.000276* |
| **CITED2** | -0.82622 | *0.003472* | -0.01795 | *0.96365* | -0.1987 | *0.263382* |
| **CMTM2** |  |  | 0.315049 | *0.419705* | 0.341669 | *0.269702* |
| **COBRA1** |  |  |  |  | 0.557292 | *1.34E-06* |
| **COPS2** | 0.3001 | *0.10285* | -0.75801 | *0.059075* | -0.36362 | *0.007725* |
| **COPS5** | 0.197706 | *0.391837* |  |  | -1.04815 | *8.70E-06* |
| **CRABP2** | 0.248433 | *0.485666* | 0.697107 | *0.083276* | 0.681285 | *0.00239* |
| **CREBBP** | -0.34991 | *0.053863* | 0.4474 | *0.273681* | 1.134861 | *5.04E-11* |
| **CRIPAK** |  |  | 0.460284 | *0.236334* | 0.68921 | *7.26E-05* |
| **CRTC1** | 0.33413 | *0.210422* |  |  | 0.275373 | *0.095962* |
| **CRTC2** |  |  | 0.153299 | *0.685758* | 1.466315 | *1.79E-20* |
| **CRTC3** | 0.220879 | *0.138089* | -0.01187 | *0.975603* | 0.318016 | *0.262604* |
| **CTBP1** | -0.6334 | *0.057406* | -0.242 | *0.541782* | 0.988859 | *0.001453* |
| **CTBP2** | -0.37192 | *0.079611* |  |  | -0.33986 | *0.039651* |
| **CTNNB1** | 0.094863 | *0.559292* | 0.539059 | *0.158653* | 0.301984 | *0.076335* |
| **CYP19A1** | 0.233342 | *0.416367* | -0.18279 | *0.644549* | 1.255223 | *1* |
| **DAP3** | 0.306865 | *0.201343* |  |  | 0.060118 | *0.613381* |
| **DAXX** | -0.32166 | *0.299211* |  |  | 0.893382 | *1.41E-08* |
| **DCAF6** | -0.51933 | *0.035361* | -0.28988 | *0.471013* | -0.55305 | *0.005317* |
| **DDX17** | 0.311809 | *0.026009* | -0.58396 | *0.132014* | -1.02498 | *0.006093* |
| **DDX20** |  |  | -0.01104 | *0.977691* | -0.27877 | *0.041177* |
| **DDX5** |  |  |  |  | -0.91795 | *7.76E-08* |
| **DDX54** |  |  | 0.041698 | *0.915791* | 0.489848 | *7.37E-07* |
| **DHX9** | 1.144982 | *0.001157* |  |  | -0.82285 | *9.38E-05* |
| **DNTTIP2** |  |  |  |  | -0.22749 | *0.017863* |
| **DUSP1** | -1.62115 | *0.000646* | -0.78559 | *0.040342* | -2.10596 | *2.89E-08* |
| **E2F1** | 0.74024 | *2.00E-05* | -0.38276 | *0.322967* | 2.157448 | *5.98E-16* |
| **E2F2** |  |  | 1.590776 | *9.70E-05* | 2.498876 | *1.12E-12* |
| **E2F3** | 1.264002 | *2.11E-06* |  |  | 0.89237 | *5.50E-07* |
| **E2F4** | 0.132047 | *0.495019* | 0.019737 | *0.960023* | 0.727572 | *1.41E-08* |
| **EDF1** | -0.15023 | *0.468649* | 0.113676 | *0.772622* | 0.257677 | *0.016148* |
| **EFCAB6** |  |  | 0.017908 | *0.963815* | 0.210888 | *0.478948* |
| **EHMT2** | -0.33171 | *0.098334* |  |  | 0.610767 | *0.00037* |
| **ELL** | -0.14143 | *0.563788* |  |  | 0.850388 | *4.79E-08* |
| **EP300** | 0.451994 | *0.021285* | 0.848797 | *0.028404* | 0.217158 | *0.249618* |
| **ESR1** | 0.558023 | *0.00918* | 0.6709 | *0.091881* | 0.284837 | *0.061548* |
| **ESR2** | 0.701262 | *0.0006* | -0.13847 | *0.724791* | -1.26989 | *3.47E-07* |
| **ESRRA** | 0.184912 | *0.469951* |  |  | 1.18075 | *4.15E-10* |
| **ESRRB** |  |  | -0.52297 | *0.188101* | -0.66718 | *0.008532* |
| **ESRRG** | -0.6265 | *0.040884* | -0.90528 | *0.023676* | 0.515943 | *0.004814* |
| **FAF1** | -0.27048 | *0.133792* | 0.178078 | *0.643465* | 0.008578 | *0.973906* |
| **FEN1** | 0.788283 | *3.84E-05* |  |  | -0.24993 | *0.279085* |
| **FHL2** | -0.87858 | *0.020785* | -0.10605 | *0.783567* | -3.0089 | *1.09E-17* |
| **FHOD1** | 0.313498 | *0.091978* | 0.528287 | *0.181616* | 0.594503 | *3.09E-05* |
| **FKBP4** | 0.846399 | *5.76E-05* |  |  | 0.779658 | *5.23E-05* |
| **FKBP5** |  |  |  |  | -0.11423 | *0.711563* |
| **FLII** | -0.17743 | *0.417297* | 1.183687 | *0.003104* | 0.006007 | *0.995147* |
| **FLNA** | -0.35295 | *0.084005* | -0.35656 | *0.352129* | -1.8706 | *4.10E-11* |
| **FOXG1** | 0.519718 | *0.056239* | 0.061537 | *0.871417* | 3.231448 | *0.068881* |
| **FOXH1** | 0.062468 | *0.78799* |  |  | 0.670545 | *1.58E-06* |
| **FOXO1** | -1.1022 | *0.002982* | -0.33868 | *0.379396* | -1.28388 | *2.58E-16* |
| **FOXO4** |  |  | -0.17801 | *0.649452* | 1.473558 | *1* |
| **FUS** | 0.635622 | *0.049681* | 0.837716 | *0.030395* | 0.107777 | *0.637129* |
| **GADD45A** | 0.044311 | *0.904556* | -0.39601 | *0.321134* | -0.53208 | *0.043813* |
| **GADD45B** | -1.01237 | *0.013614* | -0.11059 | *0.775848* | -1.20025 | *6.95E-05* |
| **GADD45G** | -0.05933 | *0.836494* |  |  | 0.256011 | *0.356767* |
| **GADD45GIP1** | -0.12466 | *0.618386* | -0.34717 | *0.373907* | 0.690155 | *4.83E-07* |
| **GATA3** | -0.46267 | *0.159623* | -0.24232 | *0.528063* | -0.30177 | *0.184219* |
| **GMEB1** |  |  | 0.109298 | *0.780903* | 0.113635 | *0.337106* |
| **GMEB2** | 0.314033 | *0.046333* | 0.51767 | *0.182611* | 0.989638 | *1.97E-07* |
| **GNB2L1** | 0.522858 | *0.011876* |  |  | -0.77294 | *4.29E-06* |
| **GSN** | 0.253733 | *0.449387* | 0.120918 | *0.75702* | -1.62263 | *2.67E-09* |
| **H2AFZ** | 0.828448 | *0.000958* |  |  | -0.74748 | *0.000162* |
| **HDAC1** | -0.50162 | *0.027751* | 0.081268 | *0.839164* | -0.08596 | *0.553242* |
| **HDAC2** | 0.128467 | *0.771798* | -0.36288 | *0.354982* | -0.08568 | *0.632316* |
| **HDAC3** | 0.204472 | *0.178163* | 0.16627 | *0.677315* | -0.12455 | *0.358311* |
| **HDAC4** | 0.838013 | *0.000697* | -0.05948 | *0.878087* | 0.255623 | *0.077028* |
| **HELZ2** |  |  | 0.273353 | *0.474439* |  |  |
| **HEY1** | 0.967577 | *1.23E-06* | 0.480212 | *0.222279* | 0.274761 | *0.24405* |
| **HHEX** | 0.503355 | *0.014458* | 0.453847 | *0.257499* | -1.1331 | *1.06E-05* |
| **HIF1A** | 0.295269 | *0.123667* | -0.43802 | *0.259331* | -0.75643 | *7.99E-05* |
| **HIPK3** | 0.106628 | *0.6608* | 0.548207 | *0.161033* | -1.84951 | *1.09E-13* |
| **HMGB2** | 1.448244 | *6.70E-10* | 0.089506 | *0.822676* | 0.575476 | *0.00596* |
| **HNF4A** | -0.22732 | *0.373536* | -0.4861 | *0.203874* | 0.235394 | *0.662922* |
| **HNF4G** |  |  |  |  |  | *1* |
| **HR** | 0.515398 | *0.023942* | -0.02695 | *0.946395* | 0.065281 | *0.693332* |
| **HSD17B1** |  |  | 0.529904 | *0.195988* | 0.350305 | *0.003214* |
| **HSD17B11** | -0.395 | *0.363262* | -0.06731 | *0.865779* | -1.34116 | *1.45E-05* |
| **HSD17B12** | -0.00979 | *0.979923* |  |  | -0.47514 | *0.174315* |
| **HSD17B13** |  |  | 0.032047 | *0.932388* | -0.24527 | *0.47208* |
| **HSD17B2** | 0.126549 | *0.652965* | -0.81757 | *0.035244* | 0.940506 | *0.043207* |
| **HSD17B4** | -0.47222 | *0.249356* |  |  | -0.75047 | *0.002644* |
| **HSD17B7** | 0.022929 | *0.910356* |  |  | 0.238166 | *0.177519* |
| **HSD17B8** | 0.194652 | *0.32178* |  |  | -0.61536 | *1.22E-06* |
| **HSD3B1** | 0.085003 | *0.601517* |  |  | 1.267926 | *0.011685* |
| **HSD3B2** | 0.635333 | *0.022433* | 0.429623 | *0.273784* | 0.515665 | *0.097303* |
| **HSPA8** |  |  |  |  | -1.37772 | *1.17E-07* |
| **IDE** | -0.50467 | *0.002336* |  |  | -1.01899 | *2.86E-10* |
| **ITGB3BP** | -0.19238 | *0.268644* |  |  | -0.53524 | *0.007124* |
| **JAZF1** |  |  | -0.77473 | *0.043063* | -2.09128 | *1.07E-12* |
| **JDP2** |  |  | 0.149941 | *0.694955* | 0.167959 | *0.444947* |
| **JMJD1C** | -0.29255 | *0.266168* | 0.162369 | *0.675994* | 1.348913 | *7.84E-10* |
| **JUN** | -1.39766 | *0.000162* | -0.41034 | *0.292131* | -0.25861 | *0.498533* |
| **KAT7** | 0.513612 | *0.003641* |  |  | -0.45795 | *0.004013* |
| **KDM1A** | -0.57679 | *0.058227* |  |  | -0.53691 | *0.000686* |
| **LATS2** |  |  | 0.193161 | *0.618402* | -0.58239 | *0.002441* |
| **LCOR** |  |  | -0.57356 | *0.140967* | 0.212117 | *0.21569* |
| **LINC00312** | 0.421357 | *0.016703* | -0.06064 | *0.877022* | -1.2975 | *6.66E-06* |
| **LMO4** | 0.09125 | *0.704796* | -0.48245 | *0.204021* | -1.6593 | *1.57E-12* |
| **MCM10** | 0.351468 | *0.070647* | 0.642525 | *0.104641* | 1.793337 | *2.16E-05* |
| **MCM2** | 0.812875 | *0.000322* | 1.095933 | *0.004772* | 0.660148 | *0.000573* |
| **MCM3** | 0.591922 | *3.80E-05* | 1.166315 | *0.003635* | 0.605308 | *6.60E-06* |
| **MCM4** | 1.286095 | *5.50E-07* | 1.689772 | *4.61E-05* | 1.611922 | *1.83E-07* |
| **MCM5** | 0.082697 | *0.564021* |  |  | 0.504036 | *0.005506* |
| **MCM8** |  |  | -0.72978 | *0.058796* | 0.75477 | *5.82E-05* |
| **MCRS1** | -0.00204 | *0.9943* | 0.548481 | *0.161154* | 0.178508 | *0.134683* |
| **MED1** | 1.807441 | *2.42E-05* | -0.5033 | *0.188934* | -0.54719 | *0.001903* |
| **MED12** | 0.621257 | *0.025644* |  |  | 0.808751 | *8.01E-08* |
| **MED14** | 0.51759 | *0.079927* | -0.24801 | *0.511215* | 0.051658 | *0.752889* |
| **MED15** | 0.196279 | *0.384767* | -0.44824 | *0.252488* | 0.581733 | *0.001127* |
| **MED24** | 0.58824 | *0.005504* | 0.503261 | *0.200294* | 0.362573 | *0.006655* |
| **MED30** |  |  | 0.226437 | *0.56574* | -0.10284 | *0.695091* |
| **MGMT** | -0.26887 | *0.16863* | -0.27785 | *0.476526* | -0.76069 | *2.61E-08* |
| **MLL2** | -0.00844 | *0.949324* |  |  | 0.485082 | *8.24E-05* |
| **MMS19** | 0.312751 | *0.135691* | -0.06485 | *0.870635* | 0.322956 | *0.00329* |
| **MNAT1** | -0.39543 | *0.221023* | 0.075012 | *0.848052* | -0.34366 | *0.027031* |
| **MPG** | 0.23271 | *0.153991* | 0.192735 | *0.62459* | -0.35167 | *0.001072* |
| **MTA1** | 1.088872 | *5.42E-08* | 1.380047 | *0.000818* | 0.979812 | *3.85E-07* |
| **MTA2** | -0.06193 | *0.703413* | 0.308107 | *0.412466* | 0.788206 | *3.97E-08* |
| **MTOR** | -0.62347 | *0.000634* | -0.35128 | *0.380704* | -0.56011 | *0.001155* |
| **MUC1** | 0.673449 | *0.219979* | 0.364671 | *0.352976* | 1.215111 | *0.001216* |
| **NCOA1** | -0.63091 | *0.000102* | -0.1443 | *0.7043* | -0.07172 | *0.621092* |
| **NCOA2** | 0.646184 | *0.006609* | 0.050348 | *0.898112* | -0.37873 | *0.050658* |
| **NCOA3** | 1.062015 | *3.56E-07* | 0.177379 | *0.642189* | 0.679251 | *4.53E-05* |
| **NCOA4** | -0.53426 | *0.00124* | -1.13232 | *0.00381* | -1.44953 | *1.01E-11* |
| **NCOA5** |  |  | 0.143226 | *0.708338* | 0.596744 | *4.15E-05* |
| **NCOA6** | 0.20332 | *0.229436* | 0.841281 | *0.037962* | -0.05649 | *0.687585* |
| **NCOR1** | 0.344879 | *0.093707* | 0.121361 | *0.751004* | 1.220552 | *7.20E-05* |
| **NCOR2** | -0.30271 | *0.269912* | 0.660308 | *0.098482* | 0.677875 | *9.17E-05* |
| **NEDD4** | -0.16599 | *0.514834* | -0.43478 | *0.274977* | -1.48908 | *1.02E-15* |
| **NELFB** | 0.397643 | *0.091525* | 0.483182 | *0.224061* |  |  |
| **NFYA** | 0.850169 | *0.00073* | 0.916231 | *0.017915* | 0.390949 | *0.004328* |
| **NFYB** | 0.45941 | *0.001455* |  |  | -0.0351 | *0.95254* |
| **NFYC** | -0.23352 | *0.25636* |  |  | 0.365683 | *0.039105* |
| **NONO** |  |  |  |  | 0.502249 | *0.004738* |
| **NQO1** | 0.385038 | *0.142267* | -0.51197 | *0.202706* | -0.87456 | *0.015686* |
| **NR0B1** | 0.101055 | *0.713748* | -0.20433 | *0.610692* | 2.511754 | *0.003086* |
| **NR0B2** | 0.570016 | *0.010344* | 0.873935 | *0.020332* | 0.733796 | *0.029284* |
| **NR1D1** | 0.598653 | *0.014491* | -0.28859 | *0.462567* | 0.457552 | *0.071626* |
| **NR1D2** | -0.06162 | *0.872051* | 0.275761 | *0.479823* | -1.11433 | *4.89E-06* |
| **NR1H2** |  |  | 0.608479 | *0.114141* | 0.675561 | *8.06E-07* |
| **NR1H3** | 0.415649 | *0.044338* | -0.40891 | *0.294291* | -0.40444 | *0.007253* |
| **NR1H4** |  |  | -0.39026 | *0.329574* | 0.705448 | *0.042551* |
| **NR1I2** | 0.034256 | *0.89957* | 0.492313 | *0.208066* | 1.793057 | *0.019392* |
| **NR2C1** | 0.363866 | *0.071157* | 0.338287 | *0.396023* | -0.01436 | *0.958293* |
| **NR2C2** | 0.497451 | *0.003726* | 0.754324 | *0.054356* | 1.239306 | *1.46E-13* |
| **NR2E1** |  |  | -0.32122 | *0.414707* | 0.221966 | *0.573227* |
| **NR2E3** | 0.146356 | *0.532625* | 0.332033 | *0.391102* | -0.08881 | *0.773401* |
| **NR2F1** | -1.59852 | *0.000102* | -0.07929 | *0.843212* | -0.52707 | *0.111632* |
| **NR2F2** | -0.78853 | *0.000325* | -0.14517 | *0.707798* | -0.29369 | *0.117803* |
| **NR2F6** | -0.17512 | *0.653708* | 1.241612 | *0.001315* | 0.358105 | *0.016566* |
| **NR3C1** | 0.315748 | *0.210353* | 0.50082 | *0.200028* | -0.65991 | *0.010368* |
| **NR3C2** | -0.43936 | *0.08381* | 0.360542 | *0.350652* | -1.04074 | *0.001438* |
| **NR4A2** | -1.4206 | *0.000912* | 0.023096 | *0.954518* | -1.352 | *2.89E-05* |
| **NR4A3** | -0.34719 | *0.143438* | -0.31284 | *0.416635* | -3.03352 | *4.39E-13* |
| **NR5A1** | 0.477616 | *0.042476* | 0.477814 | *0.221706* | 0.547254 | *0.003584* |
| **NR5A2** | 0.615663 | *0.01705* | -0.58561 | *0.148567* | 0.57609 | *0.000286* |
| **NR6A1** | 0.374488 | *0.072994* | -0.27486 | *0.475331* | 0.843075 | *0.005648* |
| **NRBF2** | 0.273534 | *0.196171* |  |  | -0.57297 | *0.002374* |
| **NRIP1** | -0.52436 | *0.055929* | 0.106061 | *0.79006* | -1.21262 | *2.57E-10* |
| **NRIP2** | 0.357964 | *0.100639* | 0.896365 | *0.02625* | -0.37907 | *0.091439* |
| **NUMA1** | -0.78747 | *0.001909* | 0.264091 | *0.496898* | -0.03579 | *0.864564* |
| **PA2G4** | 0.269039 | *0.07562* |  |  | -0.29211 | *0.007641* |
| **PADI4** | 0.495578 | *0.09967* | -0.22852 | *0.55966* | 0.890495 | *0.00109* |
| **PAK6** |  |  | 0.004749 | *0.99011* | 0.189985 | *0.521362* |
| **PARK7** | -0.5936 | *0.000923* |  |  | -0.78826 | *9.38E-07* |
| **PARP1** |  |  |  |  | 0.585227 | *2.61E-09* |
| **PDPK1** | -1.03989 | *0.000319* | -0.14 | *0.712096* | 0.048932 | *0.828554* |
| **PELP1** | -0.62883 | *0.00082* |  |  | 0.845196 | *4.90E-08* |
| **PGR** |  |  | -0.566 | *0.143523* | -3.86759 | *1.17E-23* |
| **PHB2** | -0.24145 | *0.114147* | -0.58909 | *0.13826* | -0.22258 | *0.089389* |
| **PIAS1** | 0.603136 | *0.012051* | -0.36402 | *0.347158* | 0.026202 | *0.869062* |
| **PIAS2** | -0.1396 | *0.504513* | -0.10049 | *0.797055* | 0.721273 | *0.003403* |
| **PIAS3** | 0.413151 | *0.092014* | 1.445097 | *0.000301* | 0.309855 | *0.072034* |
| **PIAS4** | 0.5934 | *0.05565* | 0.438727 | *0.252585* | 1.051366 | *0.210902* |
| **PIN1** | -0.39862 | *0.286904* | -0.11086 | *0.772837* | -0.27105 | *0.003486* |
| **PLAGL1** | -0.25197 | *0.417332* | -0.47752 | *0.220892* | -0.31459 | *0.043118* |
| **PNRC1** | -0.02027 | *0.917733* | -0.70242 | *0.069252* | -0.74931 | *0.000296* |
| **PNRC2** | -0.26295 | *0.042476* |  |  | -1.21969 | *2.98E-09* |
| **POU4F2** | 0.691921 | *0.005216* | 0.560832 | *0.161966* | 0.095437 | *0.736996* |
| **PPARA** | 0.283952 | *0.270372* | -0.36154 | *0.359027* | -0.44731 | *0.000108* |
| **PPARD** | -0.12991 | *0.75718* | 0.280793 | *0.463135* | 0.843342 | *1.67E-08* |
| **PPARG** | 0.832804 | *0.011876* | 1.187776 | *0.003977* | -0.41049 | *0.162794* |
| **PPARGC1A** | 0.028431 | *0.865211* | -0.23084 | *0.54193* | 0.407004 | *0.328024* |
| **PPARGC1B** |  |  |  |  | -0.81807 | *0.002873* |
| **PPID** | 0.082009 | *0.711852* |  |  | 1.221013 | *4.57E-07* |
| **PPM1D** | 0.256021 | *0.154243* | -0.11699 | *0.762058* | -1.23774 | *1.90E-08* |
| **PPP5C** | 0.109999 | *0.680673* | -0.04893 | *0.90416* | -0.11486 | *0.588816* |
| **PPRC1** | 0.487265 | *0.054132* | 0.186716 | *0.632746* | 0.92931 | *8.09E-08* |
| **PRAME** | 0.363755 | *0.160694* | -0.10704 | *0.775589* | 1.838044 | *0.01265* |
| **PRIC285** |  |  |  |  | 0.816993 | *9.38E-11* |
| **PRKCD** | 0.268639 | *0.451508* | 1.192062 | *0.00259* | 0.66869 | *4.34E-05* |
| **PRMT1** | 0.481783 | *0.210141* | 0.385877 | *0.333534* | -0.93932 | *3.93E-11* |
| **PRMT2** | 0.605294 | *0.001968* | -0.39994 | *0.284111* | -0.28697 | *0.010219* |
| **PRMT3** | 0.274313 | *0.145352* | 0.087351 | *0.826685* | -0.80404 | *2.79E-07* |
| **PRMT5** | -0.28916 | *0.538829* |  |  | -0.66212 | *0.0001* |
| **PRMT6** |  |  | -0.1638 | *0.685528* | -1.61929 | *1.46E-11* |
| **PROX1** | -0.1715 | *0.429835* | 0.90704 | *0.022255* | 0.717203 | *0.057842* |
| **PRPF6** | -0.2644 | *0.228632* | 0.459268 | *0.23538* | -0.0057 | *1* |
| **PSMC3** | -0.6517 | *0.111674* |  |  | -0.90534 | *2.34E-08* |
| **PSMC3IP** | 0.68098 | *0.001345* | 0.924843 | *0.022565* | 1.012512 | *3.34E-08* |
| **PSMC4** | -0.13572 | *0.761498* | 0.288478 | *0.452471* | -0.20306 | *0.08683* |
| **PSMC5** | -0.32462 | *0.180514* |  |  | -0.46102 | *0.003704* |
| **PSME3** | 0.443126 | *0.015521* | 1.015852 | *0.010751* | -0.80174 | *0.000469* |
| **PTEN** |  |  |  |  | -2.54239 | *7.49E-17* |
| **PTK2B** | -0.2902 | *0.210656* | -0.61675 | *0.118722* | 0.594587 | *0.000152* |
| **PTMA** |  |  |  |  | 0.211441 | *0.083205* |
| **PTMS** |  |  | 0.544639 | *0.166971* | 0.790832 | *1.28E-07* |
| **PUS1** | 0.10484 | *0.689286* | 0.367553 | *0.346071* | 0.825295 | *4.53E-07* |
| **PUS3** | 0.202404 | *0.244171* | -0.86393 | *0.028665* | 0.0589 | *0.736704* |
| **RAD54L2** |  |  | -0.48505 | *0.212448* | 0.62785 | *6.57E-05* |
| **RAN** | 0.44974 | *0.030843* |  |  | -0.48013 | *0.004148* |
| **RANBP9** | 0.68002 | *0.009502* | -0.38164 | *0.340877* | -0.32388 | *0.044019* |
| **RARA** | -0.37577 | *0.097436* | 0.168293 | *0.662616* | 0.429475 | *0.024548* |
| **RARB** | 0.744385 | *0.004837* | -0.05501 | *0.887583* | -2.16172 | *8.03E-13* |
| **RARG** | 0.324743 | *0.111066* |  |  | 0.631243 | *0.003825* |
| **RB1** | -0.16409 | *0.419916* | 0.080126 | *0.837869* | -0.35296 | *0.041728* |
| **RBFOX2** | -0.48025 | *0.066126* | -0.48686 | *0.220323* | -0.5435 | *0.005213* |
| **RBM14** | 0.732738 | *0.015638* | 0.624098 | *0.10528* | 1.133173 | *4.79E-06* |
| **RBM39** |  |  | -0.13846 | *0.716816* | -0.13653 | *0.30715* |
| **RCC1** | 0.790624 | *0.000572* | 0.189699 | *0.61621* | 0.759452 | *3.63E-07* |
| **RCHY1** | -0.20087 | *0.392522* | -0.03826 | *0.924443* | -1.29449 | *5.21E-08* |
| **RELA** | 0.513781 | *0.064616* |  |  | 0.744215 | *0.003445* |
| **RERE** | -0.52455 | *0.008645* | 0.79163 | *0.041568* | 0.298969 | *0.019578* |
| **RHEB** |  |  |  |  | 0.727269 | *0.000852* |
| **RNF14** | 0.279159 | *0.094252* | 0.231554 | *0.554351* | -0.43123 | *0.027628* |
| **RNF4** | -0.03597 | *0.869667* | 0.684804 | *0.069154* | -0.00996 | *0.973252* |
| **RORA** | 0.495705 | *0.032673* | -0.30183 | *0.431702* | -1.56693 | *2.72E-13* |
| **RORB** | -0.36305 | *0.253253* | -0.23267 | *0.544488* | -2.64392 | *1.69E-06* |
| **RORC** |  |  | -0.75789 | *0.053453* | 0.83055 | *4.48E-05* |
| **RPL7** |  |  |  |  | -0.18551 | *0.397258* |
| **RPL7A** |  |  |  |  | -0.18573 | *0.151265* |
| **RXRA** | -0.21402 | *0.318626* | 0.525378 | *0.172707* | 1.57437 | *3.34E-11* |
| **RXRB** | 0.367274 | *0.042623* |  |  | 0.043222 | *0.774612* |
| **RXRG** | 0.074393 | *0.795654* | -0.60546 | *0.131407* | 0.144923 | *0.687074* |
| **SAFB** | 0.299843 | *0.035704* |  |  | 1.599435 | *3.85E-11* |
| **SAFB2** | 0.084808 | *0.727145* | 0.170366 | *0.65602* | 0.844325 | *7.40E-05* |
| **SAP30** | 0.158037 | *0.479641* |  |  | -0.63319 | *0.007289* |
| **SART3** | 0.724604 | *0.000241* | 0.790063 | *0.046098* | 1.173802 | *0.451429* |
| **SATB1** | -1.22624 | *0.00609* | 0.00854 | *0.982655* | -1.74061 | *2.52E-08* |
| **SCAND1** | -0.09232 | *0.704631* | 0.347784 | *0.363663* | -0.00098 | *1* |
| **SENP1** |  |  | -0.16514 | *0.674229* | 0.411038 | *0.049888* |
| **SET** | -0.06661 | *0.794161* |  |  | 0.386161 | *0.04074* |
| **SF1** | 1.292041 | *5.51E-05* | -0.38054 | *0.336311* | -0.24365 | *0.383753* |
| **SF3A1** | -0.10487 | *0.576599* | 0.352484 | *0.369514* | -0.49019 | *0.000218* |
| **SFPQ** | 0.978099 | *9.45E-05* |  |  | 0.880673 | *2.07E-07* |
| **SGTA** | -1.20912 | *8.87E-08* | 0.366564 | *0.354395* | -0.14791 | *0.324661* |
| **SIN3A** |  |  | 0.692452 | *0.072359* | -1.12972 | *3.46E-11* |
| **SIN3B** | 0.483319 | *0.004601* | 0.181517 | *0.638759* | 0.943152 | *1.35E-06* |
| **SIRT1** | 0.31385 | *0.083249* |  |  | -0.50574 | *0.000358* |
| **SIX3** | -0.19697 | *0.088018* | -0.35308 | *0.366179* | 0.857342 | *0.001762* |
| **SKI** | -0.08051 | *0.773946* | 0.187073 | *0.624253* | 0.951828 | *5.55E-05* |
| **SLIRP** | 0.563288 | *0.02061* |  |  | 0.008137 | *0.988812* |
| **SMAD3** | 0.586283 | *0.024124* | -0.00425 | *0.991284* | -0.02321 | *0.940066* |
| **SMAD4** | -0.10847 | *0.685589* | -0.27644 | *0.482675* | -1.52759 | *2.47E-12* |
| **SMARCA2** | -0.38099 | *0.112985* | -0.74696 | *0.051904* | -1.15744 | *4.00E-09* |
| **SMARCA4** | -0.61811 | *0.012114* | 0.862269 | *0.027345* | 0.31861 | *0.183724* |
| **SMARCD1** | 0.335535 | *0.287004* | 0.863002 | *0.026374* | 0.806865 | *1.24E-08* |
| **SMARCD3** | -0.59351 | *0.029027* | -0.16863 | *0.665371* | -0.37636 | *0.09335* |
| **SMARCE1** |  |  | -0.13236 | *0.729735* | -1.90158 | *4.50E-12* |
| **SNW1** | -0.25682 | *0.23181* | 0.330225 | *0.39391* | -0.23254 | *0.02208* |
| **SOX4** | 0.460982 | *0.159074* | 0.526685 | *0.182332* | 0.105057 | *0.754117* |
| **SPDEF** | -1.21396 | *6.69E-07* | -0.11167 | *0.774431* | -0.88995 | *1.98E-05* |
| **SPEN** | 0.500144 | *0.009157* | 0.63217 | *0.106163* | 0.372271 | *0.004074* |
| **SQSTM1** | 0.171696 | *0.232753* | 0.087011 | *0.822855* |  | *1* |
| **SRA1** | 0.408431 | *0.014477* | -0.37831 | *0.329422* | -0.33459 | *0.012308* |
| **SRCIN1** |  |  |  |  | 1.328615 | *2.39E-05* |
| **SRD5A1** | 1.090058 | *9.42E-05* |  |  | 0.178394 | *0.596282* |
| **SRD5A2** | -0.79842 | *0.005249* | -0.76701 | *0.045146* | -5.00464 | *1.36E-28* |
| **SREBF1** | 0.103977 | *0.636937* | 0.156922 | *0.689427* | 0.335976 | *0.053825* |
| **SREBF2** | -0.17224 | *0.59451* | 0.145033 | *0.708652* | 1.019298 | *5.16E-12* |
| **SRY** |  |  |  |  | 0.361545 | *0.557142* |
| **SS18** | 0.443207 | *0.060826* | -0.43644 | *0.268872* | 0.010971 | *0.962387* |
| **STAT3** | -0.6325 | *0.011178* | 0.799917 | *0.044655* | -0.31942 | *0.240753* |
| **STAT5A** | 0.453691 | *0.102984* | -0.18409 | *0.634607* | -0.63897 | *0.000106* |
| **STAT5B** | 0.305395 | *0.191107* | -0.18363 | *0.628277* | 0.701459 | *2.03E-05* |
| **STS** | 0.550322 | *0.03395* |  |  | -0.69718 | *0.001685* |
| **STX8** |  |  | -0.5933 | *0.121545* | -0.58871 | *4.16E-06* |
| **SUB1** | 0.54231 | *0.001403* |  |  | -0.36084 | *0.039239* |
| **SULT1E1** | 0.112091 | *0.559444* |  |  | 1.472591 | *0.069006* |
| **SUMO1** |  |  | 0.231975 | *0.550891* | -0.51617 | *0.005126* |
| **SUPT6H** | -0.52517 | *0.090653* | -0.18962 | *0.623193* | -0.21074 | *0.272964* |
| **SUPT7L** | 0.448268 | *0.004418* |  |  | -0.39095 | *0.106836* |
| **SVIL** | -0.08626 | *0.747562* | 0.512077 | *0.181566* | -1.60973 | *1.34E-12* |
| **TADA2A** | 0.801646 | *7.84E-05* | -0.65392 | *0.101772* | 0.081554 | *0.682702* |
| **TADA3** | -1.86629 | *1.79E-06* | -0.22069 | *0.576368* | 0.509281 | *0.027749* |
| **TAGLN** | -1.92451 | *1.13E-05* | -0.27435 | *0.483797* | -1.88294 | *6.99E-13* |
| **TBL1X** | 0.339095 | *0.108239* | -0.017 | *0.964588* | 0.587315 | *0.001138* |
| **TBL1XR1** |  |  |  |  | -0.05509 | *0.81155* |
| **TCF20** | 0.609076 | *0.000645* | 0.204839 | *0.589693* | 1.187864 | *8.80E-11* |
| **TCF21** | -1.27624 | *1.18E-06* | -0.36384 | *0.359889* | -2.32093 | *5.90E-21* |
| **TDG** | 0.802754 | *0.000113* |  |  | 0.208543 | *0.137209* |
| **TGFB1I1** | -0.12532 | *0.617751* | -0.39925 | *0.298022* | -1.33498 | *5.07E-10* |
| **TGIF1** | -0.61752 | *0.01079* | 0.564165 | *0.148309* | 0.362708 | *0.025007* |
| **TGS1** | 0.28601 | *0.048449* |  |  | -0.16141 | *0.331796* |
| **THRA** | 0.43343 | *0.065742* | 0.124948 | *0.749812* | -0.0551 | *0.757715* |
| **THRB** | 0.099958 | *0.744554* | -0.43364 | *0.287995* | 0.327497 | *0.354103* |
| **TMF1** | 0.063349 | *0.785529* | 0.144497 | *0.707752* | 0.313758 | *0.116981* |
| **TP53** | -0.19108 | *0.441698* |  |  | -0.85153 | *0.000414* |
| **TPX2** | 1.942114 | *1.05E-08* | 2.182146 | *8.20E-08* | 2.446769 | *3.42E-13* |
| **TRIM24** | 0.607213 | *0.042743* | 0.512382 | *0.042026* | 0.509293 | *0.049764* |
| **TRIM25** | 0.134936 | *0.630369* |  |  | 0.391034 | *0.087968* |
| **TRIM28** | 1.13074 | *3.84E-05* | 0.71547 | *0.039095* | 0.739373 | *5.49E-06* |
| **TRIP10** | 0.143589 | *0.545098* | 0.131007 | *0.734853* | 0.434656 | *0.010081* |
| **TRIP11** | 0.586453 | *0.029097* |  |  | 0.273329 | *0.151345* |
| **TRIP12** | 0.173485 | *0.516313* | 0.031983 | *0.936869* | -0.91312 | *1.76E-05* |
| **TRIP13** | 0.859433 | *3.27E-06* | 1.004512 | *0.011166* | 1.996201 | *9.49E-05* |
| **TRIP4** |  |  | -0.20309 | *0.602532* | -0.27859 | *0.002528* |
| **TRIP6** | -0.33449 | *0.146064* |  |  | -0.85745 | *6.29E-06* |
| **TRRAP** | -0.56417 | *0.001919* | 0.706207 | *0.069621* | 0.864006 | *8.29E-12* |
| **TSC2** | 0.244303 | *0.288841* | 0.4794 | *0.217712* | 0.694759 | *5.65E-06* |
| **TXN** | 0.636954 | *0.002218* |  |  | 0.158987 | *0.414539* |
| **TXNRD2** | -0.47854 | *0.190107* | -0.2179 | *0.580776* | -1.32771 | *5.55E-09* |
| **UBE2I** | 0.080518 | *0.74636* | 0.053242 | *0.8898* | -0.21578 | *0.306689* |
| **UBE2L3** | -0.49648 | *0.000153* | -0.39071 | *0.330884* | -0.89556 | *7.70E-10* |
| **UBE3A** | 0.467857 | *0.028121* | 0.033681 | *0.929973* | 0.012869 | *0.958293* |
| **UBR5** | 0.935131 | *6.69E-07* | 0.355459 | *0.370994* | 0.338521 | *0.069614* |
| **UGT2B11** |  |  |  |  | 1.204134 | *0.022655* |
| **UIMC1** |  |  |  |  | 0.479457 | *2.35E-06* |
| **UNC45A** | -0.17969 | *0.255165* | -0.33899 | *0.396239* | -0.78668 | *3.35E-05* |
| **UXT** | 0.18275 | *0.459388* | -0.52127 | *0.174531* | -0.25847 | *0.060364* |
| **VAV3** | 0.506853 | *0.069744* | 0.099892 | *0.793134* | 1.051794 | *0.004401* |
| **VDR** | 1.480533 | *2.97E-06* | 0.154195 | *0.690007* | 0.904918 | *0.000609* |
| **WDR77** | -0.09725 | *0.660311* |  |  | 0.018858 | *0.921546* |
| **XBP1** |  |  |  |  | -1.65464 | *5.96E-12* |
| **XRCC5** | 0.372287 | *0.017311* | 0.277455 | *0.478433* | -0.38341 | *0.012095* |
| **XRCC6** | -0.3388 | *0.017669* |  |  | -1.37728 | *1.45E-12* |
| **YWHAH** | 0.422008 | *0.015508* | 0.544641 | *0.168571* | -0.03449 | *0.882068* |
| **ZFPM2** | 0.278931 | *0.145657* | -0.05241 | *0.896734* | 1.46266 | *3.58E-06* |
| **ZMIZ1** | 0.149422 | *0.589606* | 1.220054 | *0.002505* | 0.850467 | *3.10E-07* |
| **ZMIZ2** | 0.421807 | *0.019582* | 0.261134 | *0.500545* | 1.481609 | *2.08E-11* |
| **ZNF318** | 0.415241 | *0.012021* | -0.08095 | *0.837905* | 1.210035 | *6.53E-07* |
| **ZNF461** |  |  |  |  | -0.03207 | *0.890348* |
| **ZNF653** |  |  | 0.009725 | *0.980039* | 0.892439 | *1.82E-07* |
| **ZNHIT3** | -0.05463 | *0.735095* | 0.093855 | *0.807565* | -0.79929 | *8.52E-06* |
| **ZYX** | -0.34441 | *0.180214* | -0.01659 | *0.965622* | 0.090803 | *0.652788* |

Supplementary Table 2: List of coregulator/cofactors and their log2 fold expression changes (LFC) and adjusted p values in three CRPC cancer cohorts (GSE33269, GSE70770, GSE35988) comparing CRPC to naïve/nonCRPC samples.
